# Rapid Internalization of Nanoparticles by Human Cells at the Single Particle Level

**DOI:** 10.1021/acsnano.3c01124

**Published:** 2023-08-29

**Authors:** Ceri J. Richards, Thomas C. Q. Burgers, Rifka Vlijm, Wouter H. Roos, Christoffer Åberg

**Affiliations:** † Pharmaceutical Analysis, Groningen Research Institute of Pharmacy, University of Groningen, 9713 AV Groningen, The Netherlands; ‡ Molecular Biophysics, Zernike Institute for Advanced Materials, University of Groningen, 9747 AG Groningen, The Netherlands

**Keywords:** endocytosis, residence time, confocal microscopy, STED microscopy, human embryonic kidney cells, polystyrene nanoparticles

## Abstract

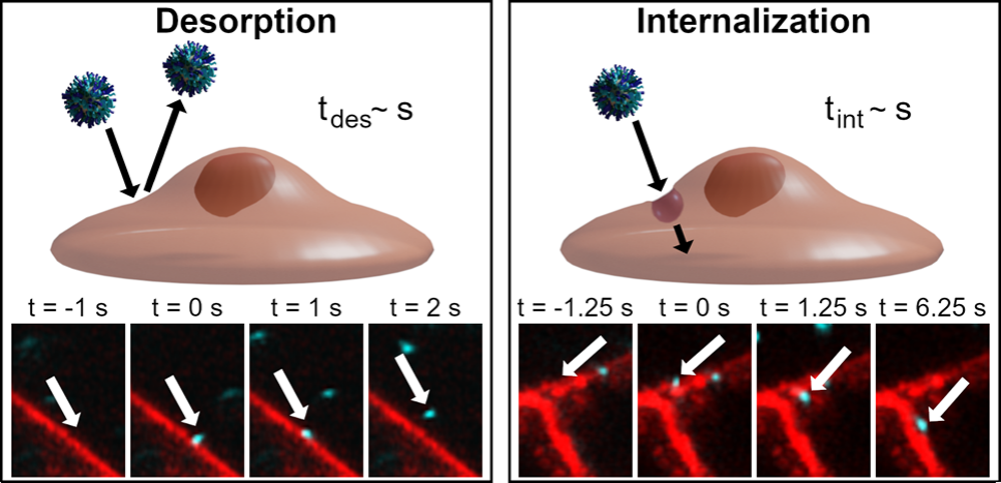

Nanoparticle uptake by cells has been studied for applications both in nanomedicine and in nanosafety. While the majority of studies have focused on the biological mechanisms underlying particle internalization, less attention has been given to questions of a more quantitative nature, such as how many nanoparticles enter cells and how rapidly they do so. To address this, we exposed human embryonic kidney cells to 40–200 nm carboxylated polystyrene nanoparticles and the particles were observed by live-cell confocal and super-resolution stimulated emission depletion fluorescence microscopy. How long a particle remained at the cell membrane after adsorbing onto it was monitored, distinguishing whether the particle ultimately desorbed again or was internalized by the cell. We found that the majority of particles desorb, but interestingly, most of the particles that are internalized do so within seconds, independently of particle size. As this is faster than typical endocytic mechanisms, we interpret this observation as the particles entering via an endocytic event that is already taking place (as opposed to directly triggering their own uptake) or possibly via an as yet uncharacterized endocytic route. Aside from the rapidly internalizing particles, a minority of particles remain at the membrane for tens of seconds to minutes before desorbing or being internalized. We also followed particles after cell internalization, observing particles that appeared to exit the cell, sometimes as rapidly as within tens of seconds. Overall, our results provide quantitative information about nanoparticle cell internalization times and early trafficking.

Nanoparticles have been adopted by the medical field as drug delivery vehicles,^1−3^ and more recently as vaccines.^4−7^ For drug delivery, nanoparticles offer several advantages over conventional methods, such as reduction in drug degradation, inherent cancer targeting, and cell-specific targeting.^1,8−11^ In addition, the ability to engineer the properties of the carrier offers options to address specialized purposes,^12^ and, in general, affect particle-cell interactions. In particular, particle entry into the cell, which typically occurs via endocytosis,^13,14^ is a crucial step for drug delivery. Consequently, understanding how particle properties affect particle internalization^15−20^ is highly important for nanoparticle design strategies.

Despite a vast literature on the subject, there are few general outcomes about the factors that govern nanoparticle uptake.^8,13,21−23^ This lack is, in part, due to the complexity of interactions present at the cell membrane,^21,24^ and also the approaches used to interrogate particle uptake.^14,25^ Previous studies have used techniques such as inhibitors, gene silencing and overexpression.^15,26−28^ However, results from methods that alter cell functioning sometimes need to be interpreted with caution.^13,28,29^ Furthermore, such approaches typically offer information about the endocytic pathways in action but do not resolve the processes prior to endocytosis. On the other hand, quantitative spatiotemporally resolved studies can assess the dynamics of the entire nanoparticle-cell interaction and therefore shed light on factors (for example, particle adsorption or internalization rates, times, and forces) that influence it.^25,30−34^ Moreover, single particle studies can resolve subpopulations of particles that exhibit different and potentially atypical behavior, which would likely not be resolved by bulk techniques.^31,35^

The processes leading up to and including the point of particle internalization are, by necessity, mediated by interactions at the outer cell membrane. Broadly speaking, these interactions result in the adsorption of the particle onto the cell, followed by the particle remaining at the membrane, and culminate in either the desorption or the internalization of the particle.^36,37^ In order to capture the dynamics of particles in the vicinity of and on cells, spatiotemporally resolved techniques must be used. Such studies have revealed various diffusive regimes, indicating regions of differing particle-cell interactions, as well as kiss-and-run type events and particle hopping.^30,38−40^ Moreover, some studies have shown that properties such as particle size and functionalization can affect particle adsorption and desorption dynamics.^38,41−44^ For the internalization process, typical particle internalization times ranging from one to several minutes have been reported.^31,33,34,39,40^ Supported by visualization of pit formation,^31,45^ these time scales are consistent with expectations that particles bind to cell receptors, the membrane invaginates and the particle is internalized,^36,46^ a process which takes 30 s to several minutes for pathways such as clathrin-mediated endocytosis and phagocytosis.^47−49^ However, studies using pair correlation analysis have reported much shorter internalization times (≤ 0.5 s).^20,50^ It is not clear whether there is a real discrepancy between these two time scales or whether it reflects, e.g., different particle characteristics.

Here we present quantitative data at the single particle level on how long particles remain on the membrane before being internalized or desorbing. We use live-cell confocal microscopy to follow nanoparticles that adsorb onto the cell membrane and observe whether they subsequently desorb or are internalized. We support these observations with super-resolution imaging using live-cell STimulated Emission Depletion (STED) microscopy.^51^ To understand how the processes are affected by particle size, we use particles with diameters of 40, 100, and 200 nm.^16−18,35^ For all particle sizes investigated, the majority of particles desorbs from cells rapidly. Moreover, internalization occurs within just a few seconds of membrane binding. We suggest that the rapid internalization, which is much quicker than expected for endocytic mechanisms, stems from the particles entering via an endocytic event that is already taking place (as opposed to triggering their own uptake) or via an as yet uncharacterized endocytic route. We also followed the motion of the particles qualitatively while on the membrane and within the cell. Here a variety of behavior was observed, including, next to entry, also diffusion along the cell membrane and apparent exit.

## Results and Discussion

### Model System

We used 40, 100, and 200 nm (nominal diameter) carboxylated polystyrene particles as a model system because they are well characterized and exhibit bright fluorescence. Furthermore, we have previous data on their interactions with cells, showing that they are readily internalized by cells^41,52,53^ via multiple mechanisms^26^ and with a particle size-dependent uptake rate,^18,54^ and that they subsequently distribute intracellularly with a significant portion accumulating in lysosomes.^32,55^ For the cell experiments, the particles were always dispersed in medium supplemented with serum to ensure the formation of a biomolecular corona on the particle surface.^56^ This preparation method achieves complete coverage of the carboxylated polystyrene surface, as we have shown previously.^35^ Basic physicochemical characterization of the particle dispersions was performed using dynamic light scattering (size) and laser Doppler velocimetry (ζ potential) and showed results in line with previous studies (Table S1 and Figures S1 and S2 in the Supporting Information).^18,41,54,55^ Moreover, the particles are stiff and therefore expected to maintain their shape during adsorption to the cell membrane and internalization.^57−59^

As a cell model, we chose human embryonic kidney (HEK) cells as their bulky shape and limited number of membrane projections allowed for easier membrane identification compared to other cell lines. The cells were stained with a fluorescent membrane dye to visualize the outer cell membrane, and a low-concentration nanoparticle dispersion was subsequently exposed to the cells. The cells were observed by time-lapse confocal imaging, leaving the particle dispersion with the cells. Thereby nanoparticles could be visualized continuously as they adsorbed onto the cell membrane and subsequently desorbed back into dispersion or were internalized. We cannot easily differentiate between single particles, dimers, or other (loose or irreversibly bound) particle agglomerates using confocal microscopy. However, since the particles do not agglomerate uncontrollably (Table S1 and Figures S1 and S2) the majority of objects we observe are expected to be single particles, especially outside cells and just after internalization (where we will focus).

Particle exposure and microscopy affected neither cell integrity (Figure S3 in the Supporting Information) nor particle uptake rate (Figure S4 in the Supporting Information). Moreover, desorption and internalization events were present across the entire experiment, and thus, the events reported herein are not artifacts induced by continuous imaging (Figure S5 in the Supporting Information). Lastly, kinetics studies show that particle uptake proceeds throughout the time scales of our measurements (5–60 min) and for all particle sizes (Figure S6 in the Supporting Information). This observation indicates that there is no relevant saturation mechanism at these time scales, consistent with previous studies on the same particles in other cell lines.^18,41,53^

### Definition of Desorption and Internalization Events

**Figure 1:**
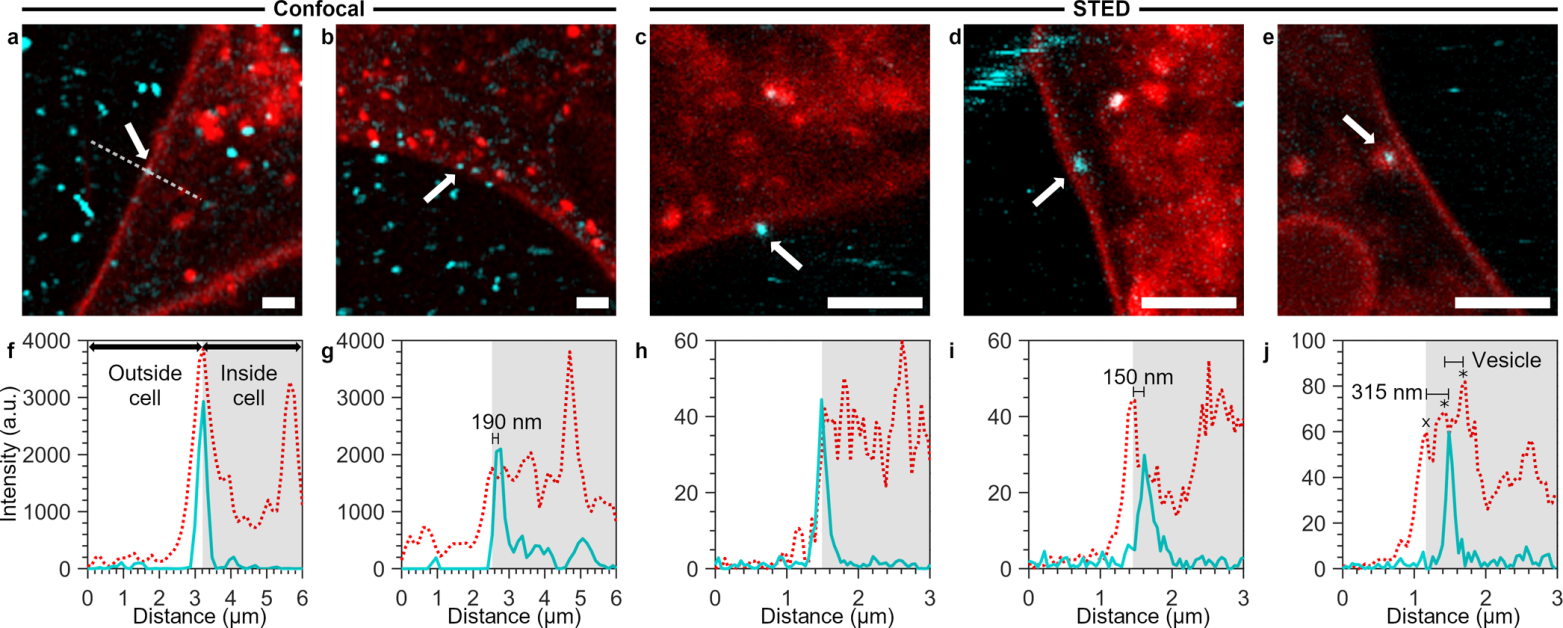
Visualization of particles adsorbing to and being internalized by cells. HEK cells were labeled with a cell membrane stain, subsequently exposed to the fluorescent particles, and observed under the microscope with the particle dispersion still present. (a, b) Confocal microscopy images of HEK cells stained with CellMask Orange (red) in the presence of yellow–green 40 nm nanoparticles (cyan): (a) adsorption event and (b) internalization event. The particles of interest are indicated with white arrows. (f–g) Line intensity profiles of the particles shown in panels (a) and (b), respectively, along a line going from outside of the cell toward the cell interior [an example of where the line was taken is shown by the white dashed line in panel (a)]. The membrane stain signal is given by the red dotted line, while the nanoparticle signal is given by the cyan solid line; the inside of the cell is indicated by the light gray shading. In panel (f), the particle intensity peak overlaps with the membrane intensity peak, indicating that the particle is adsorbed to the outer cell membrane. Conversely, in panel (g), the particle intensity peak is displaced by ∼190 nm from the membrane peak toward the cell interior, indicating that the particle has been internalized (note that the second, higher, peak at ∼4.75 μm is a vesicle inside the cell and not the outer cell membrane). (c–e) Live-cell STED microscopy images of HEK cells stained with the membrane marker Abberior Star 580-DPPE (red) in the presence of dark red 40 nm nanoparticles (cyan). (c) Particle adsorbed to the cell membrane. (d) Internalized particle. (h–i) Line intensity profiles corresponding to the particles shown in panels (c) and (d), respectively. With the improved resolution of STED, the internalized particle can be distinguished within just ∼150 nm of the cell interior. (e) Particle within a vesicle-like structure. (j) Line intensity profile corresponding to the particle shown in panel (e). From the intensity profile, the outer cell membrane peak (x) can be distinguished from the vesicle membrane peaks (*) within which the particle resides. The vesicle is pinched off from the outer cell membrane, confirming that the particle is internalized, with a particle-cell membrane peak separation of ∼315 nm. Scale bars = 2 μm.

We started by observing events in three dimensions but in a relatively thin volume by repeatedly imaging the same three *z*-planes (in total, ∼0.9 μm thick) every ∼15 s. Considering the minute-time scale internalization times associated with typical particle uptake pathways,^47−49^ this temporal resolution seemed sufficient to capture internalization events. Indeed, particles adsorbing onto the outer cell membrane could be observed (Figure 1a). In the axial (*z*) direction, the resolution in the nanoparticle channel is better than that in the membrane channel (due to the nanoparticle excitation wavelength being shorter), so the axial position of the particle is within the range of positions where the membrane is detected. Furthermore, we only considered events happening at places where the membrane is well-defined. The particles were tracked across all *z*-planes so that displacements in the *z*-direction as well as in the *x*–*y* plane could be followed. To characterize the position in the lateral (*x*–*y*) direction, we examined the fluorescence intensity along a line perpendicular to the membrane (Figure 1a, dotted line). The peaks in the membrane and particle fluorescence intensity overlap (Figure 1f), confirming that such particles really were adsorbed. It should be noted that, due to the limited temporal resolution, it is possible that particles repeatedly desorb and adsorb onto the cell membrane between each measurement frame, a process that would not be captured. Therefore, the adsorption events represent particles that interacted sufficiently strongly with the cell membrane that they did not move away from the membrane appreciably and consequently appear adhered in succeeding images.

Some of the adsorbed particles subsequently entered the cell (Figure 1b; further examples in Videos S1, S2, and S3 in the Supporting Information). To characterize the moment a particle was internalized, we again considered the fluorescence intensity profile along a line perpendicular to the membrane. Thus, we designated the particle as internalized when the fluorescence intensity profile showed a distance of at least 2 pixels (240 nm) between the maximum in the particle intensity and the maximum in the membrane intensity. Since both the particle and the membrane will actually be somewhere within either pixel, we estimate that the actual distance into the cell will be at least 1.5 pixels, amounting to 180 nm or more (as a comparison, the localization precision was determined to be 12.0 ± 0.6, 13.7 ± 0.8, and 51 ± 2 nm for the 200, 100, and 40 nm particles, respectively). We made this conservative choice to avoid incorrectly identifying a particle as having been internalized, when it actually resides in a vesicle which has not yet bud off into the cell from the cell membrane, given vesicle sizes for typical endocytic pathways such as caveolin or clathrin-mediated endocytosis of 50 and 100 nm, respectively.^47,60^ Indeed, using this criterion is consistent with visual inspection of a particle inside (Figure 1b) and fluorescence intensity profiles that are well-separated (Figure 1g). Furthermore, in some cases, we followed the particle moving several micrometers into the cell interior (see the discussion related to Figure 5 below), leaving no doubt that it was internalized.

As further evidence of our approach, we also imaged particles interacting with living cells using super-resolution STED microscopy with imaging speeds ranging from 0.5–7 s per frame (depending on the dimensions of the region imaged). As with confocal microscopy, particles that were adsorbed to the cell membrane (Figure 1c) had an overlapping nanoparticle and membrane peak intensity (Figure 1h). Moreover, particles that visually appeared clearly within the cell (Figure 1d) could be observed to exhibit particle–membrane separations smaller than 180 nm. Furthermore, particle-containing vesicles formed from the outer cell membrane were sometimes also stained by the membrane dye (Figure 1e). In this case, the fluorescence intensity peak from the outer cell membrane can be distinguished (Figure 1j, cross) from the two peaks corresponding to the two sides of the vesicle (Figure 1j, asterisks) containing the particle. From the fact that the outer membrane peak and the first peak of the vesicle are separated and that we see no evidence of a tether or neck still attached to the membrane, we conclude that the vesicle is indeed pinched off from the outer cell membrane. This presents a clear example of a completed nanoparticle internalization event, captured within just a few hundreds of nanometers from the outer cell membrane. We observed internalized particles both with (Figure 1e) and without (Figure 1d) an associated (stained) cell membrane structure, but regardless, the vast majority of particles were in vesicles (as we show below in Figure 5).

### Particle Desorption and Internalization on the 15 s to Minute Time Scale

**Figure 2:**
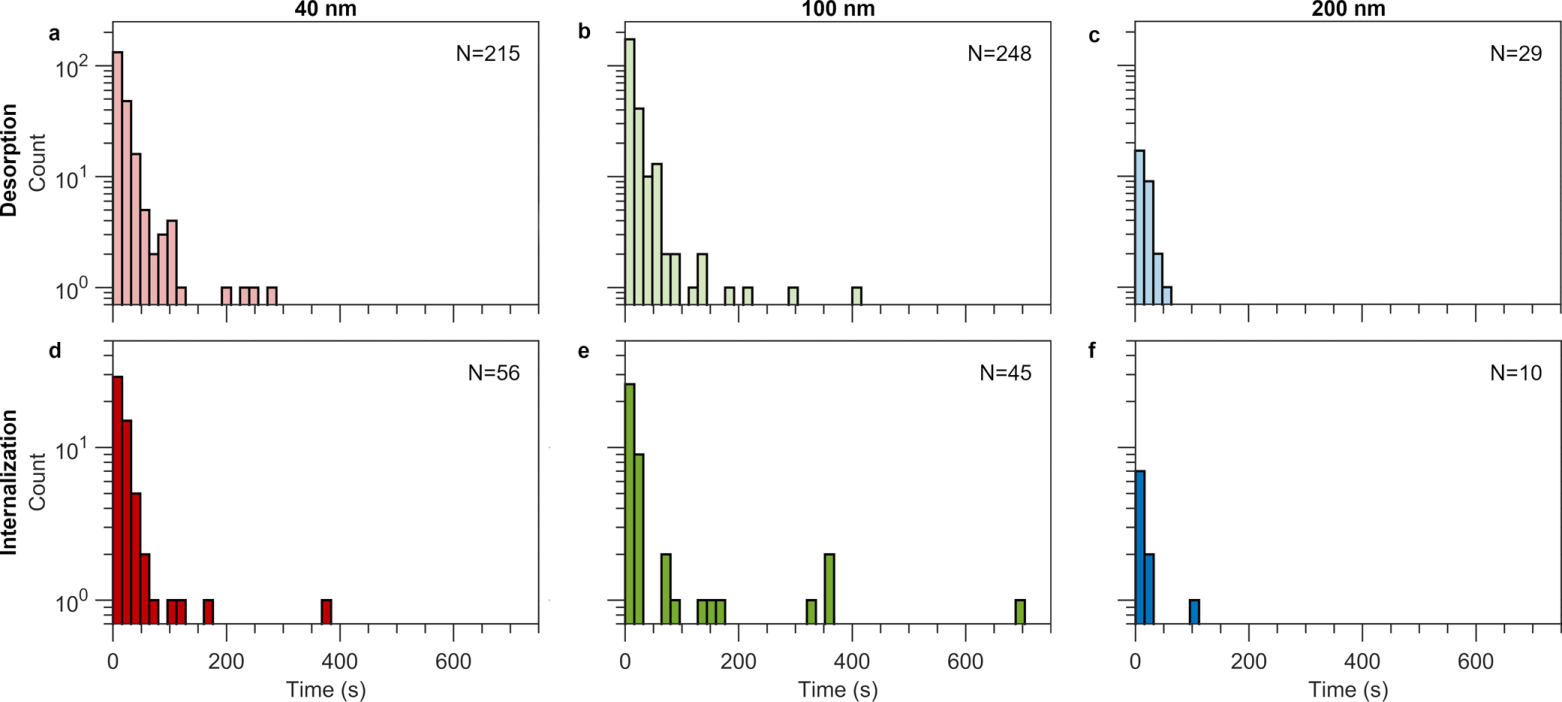
Particle desorption and internalization times for the three particle sizes. Particles adsorbing to the outer cell membrane of HEK cells were observed (as in Figure 1) and the time the particle spent at the membrane was subsequently recorded, together with whether the particle desorbed into the medium again or was internalized into the cell. (a–c) Histograms of particle desorption times for (a) 40 nm, (b) 100 nm, and (c) 200 nm particles. (d–f) Histograms of particle internalization times for (d) 40 nm, (e) 100 nm, and (f) 200 nm particles. For both the desorption and internalization events, the majority of particles were adsorbed for < 60 s for all particle sizes; for the 40 and 100 nm particles, one additionally observes a few singular events at time scales on the order of minutes. The number of events the data is based on is indicated in the graphs. We analyzed 6, 11, and 13 cells for 40, 100, and 200 nm particles, respectively. Histogram bin size: 16 s. Note the *y* logarithmic axis.

Having confirmed the approach, we observed cells using confocal microscopy and noted all particles that adsorbed to the cell membrane and for how long they remained there before either desorbing again or being internalized. We repeated this procedure for the 40, 100, and 200 nm particles, in total observing more than 600 events. We thus present the results in terms of the full distribution of desorption and internalization times (Figure 2), thereby providing quantitative information on event time scales, at the single particle level.

For the particles that desorb, we observe that, for all sizes, the majority of particles (91%, 90%, and 97% for 40, 100, and 200 nm particles, respectively) desorbed within 1 min after attachment to the membrane (Figure 2a–c). In addition, the 40 and 100 nm nanoparticles also exhibit some events at longer time scales (longer than 1 min), but these events are rarer (9% and 10% for 40 and 100 nm particles, respectively). While we observed only a single minute-long desorption event for the 200 nm particles, this could simply be due to us capturing far fewer events in general for these particles (at least partly due to using the same mass concentration). To directly compare the desorption events for the different nanoparticle sizes, we extracted a characteristic time from fits to the data (Table 1). We observe that the characteristic desorption event occurs within 12–20 s (1–2 frames) after adsorption to the cell membrane, and that this is independent of particle size. Using the mean time, which is more sensitive to the rare longer time scale events, shows the same outcome (Table S2 in the Supporting Information).

**Table 1:** Characteristic Desorption and Internalization Times for the 3D Experiments^a^

particle	characteristic desorption time (s)	characteristic internalization time (s)
40 nm	16	20
100 nm	12	13
200 nm	19	12

a To characterize the distributions (Figure 2) we fitted the equation *N*(*t*) = *N*_0_ exp(−*t*/τ), where τ is the characteristic time, to the data. As an alternative, we also used the mean time (Table S2 in the Supporting Information), but this measure is sensitive to outliers.

We also investigated cell internalization times (Figure 2d–f) and generally speaking observed the same behavior as for desorption. Thus, the majority of particles that were internalized entered the cell within 1 min after adsorbing to the cell membrane (89%, 78%, and 90% for 40, 100, and 200 nm particles, respectively), although some longer events were also observed (for the 200 nm particles, only 10 internalization events were observed but the data follow the same trend). Compared to the time scales expected for endocytosis triggered by particle binding,^47−49^ the majority of events we observe are consequently relatively short. Indeed, the characteristic internalization times remained within 20 s for all particle sizes (Table 1). In terms of the longer time scale (>1 min) events, while rare, we observed a higher proportion of these for the 100 nm particles (22%), compared to the 40 nm particles (11%); for the 200 nm particles, there are too few events to make a comparison. Therefore, while the typical internalization time is similar for the 40 and 100 nm particles, there may be more subtle size differences in terms of the longer lasting events. It should also be noted that some particles remained adsorbed to the membrane at the end of the observation time and therefore it is possible that a population of internalization or desorption events with time spans greater than ∼10 min exists, but were not observed in our experiments.

Finally, we note that comparison of the absolute number of events between particle sizes is inconsequential, because we applied the same mass (as opposed to number) concentration for all particle sizes, because there is a large cell-to-cell variability in nanoparticle uptake,^61,62^ and because we observed varying, but few, numbers of cells (6, 11, and 13 cells for 40, 100, and 200 nm particles, respectively). The distributions and the *relative* number of desorption and internalization events, however, carry meaning.

### Particle Desorption and Internalization on the Seconds Time Scale

**Figure 3:**
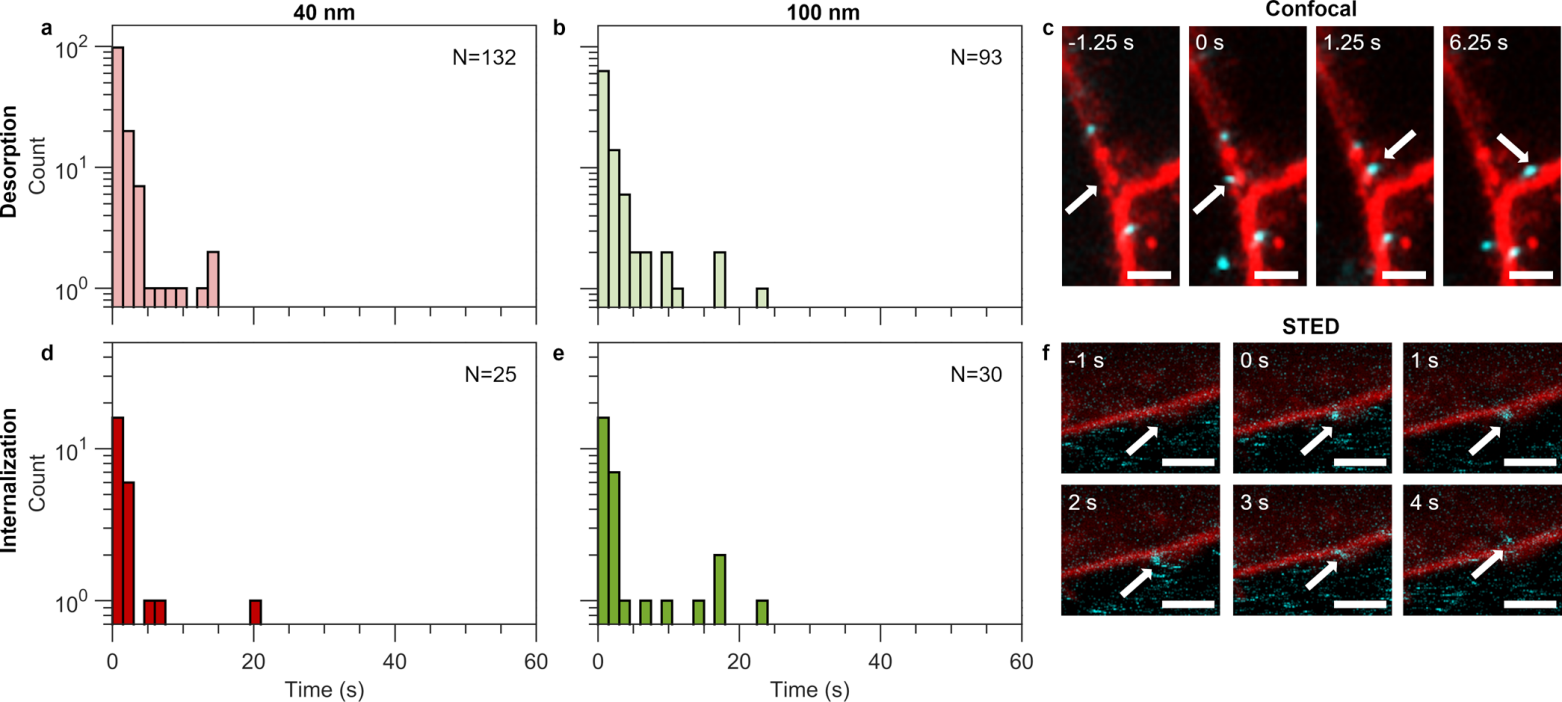
Particle desorption and internalization times at shorter time scales (≲ 1.5 s). The time particles spent at the outer HEK cell membrane before desorbing or being internalized was quantified as in Figure 2, but now at shorter time scales (∼1.5 s instead of ∼15 s). (a, b) Histograms of particle desorption times for (a) 40 nm and (b) 100 nm particles. (d, e) Histograms of particle internalization times for (d) 40 nm and (e) 100 nm particles. The majority of particles was adsorbed for less than 5 s. Histogram bin sizes of 1.5 s; 11, 14, and 17 cells were measured for 40, 100, and 200 nm particles, respectively. Note the *y* logarithmic axis and the different *x*-axis compared to Figure 2. (c) Confocal microscopy images of a particle internalization event (particle indicated by white arrows). At *t* = −1.25 s, no particle is observed. The particle is first visualized adsorbed onto the cell membrane at *t* = 0 s; it is internalized into the cell in the subsequent time frame (*t* = 1.25 s) and transported further into the cell in the following time frames (exemplified for *t* = 6.25 s). This event is also shown in Video S4 in the Supporting Information. (f) Live-cell STED images of a particle (indicated by the white arrows) first visualized adsorbed onto the cell membrane at *t* = 0 s and internalized into the cell by time frame *t* = 4 s. This event is also shown in Video S5 in the Supporting Information. Scale bars = 2 μm.

To investigate the fast events in further detail, we performed two-dimensional (2D) confocal microscopy as this allows an improved temporal resolution (1–1.5 s), but otherwise recorded desorption and internalization events in the same way (Figure 3). For the particles that desorbed (Figure 3a,b), we observed that the majority of 100 and 40 nm particles were adsorbed to the membrane for just a few seconds before desorbing (95% and 92% of the 40 and 100 nm particles, respectively, desorbed within 5 s). We made the same observation for the 200 nm particles, but the distribution is not informative since we only observed 28 events for these particles. The characteristic time scales are shown in Table 2 for all particle sizes, showing that particles typically desorbed within 3 s (Table S3 in the Supporting Information shows mean time scales).

**Table 2:** Characteristic Desorption and Internalization Times for the 2D Experiments^a^

particle	characteristic desorption time (s)	characteristic internalization time (s)
**HEK Cells**		
40 nm	1	1
100 nm	1	2
200 nm	2	—
**MDA-MB-231 Cells**		
100 nm	0.7	1.3

a To characterize the distributions (Figure 3), we fitted the equation *N*(*t*) = *N*_0_ exp(−*t*/τ), where τ is the characteristic time, to the data. As an alternative, we also used the mean time (Table S3 in the Supporting Information), but this measure is sensitive to outliers.

Interestingly, when we investigated particle internalization using the faster approach (Figure 3d,e), we saw that, for the 40 and 100 nm particles, the majority of internalization events occurred within just a few seconds of adsorbing to the cell membrane (92% and 80% of the 40 and 100 nm particles, respectively, internalized within 5 s; Table 2 and Table S3 in the Supporting Information show corresponding typical internalization times). For the 200 nm particles, we observed only four internalization events, three of which occurred within 1.5 s and one within ∼12.5 s. As an example, Figure 3c shows the rapid internalization of a 100 nm particle captured with confocal microscopy. The particle adsorbs to the membrane for a single frame (1.25 s) before it appears within the cell. Following the particle across time, it is transported further into the cell interior, showing that the particle was really internalized (Video S4 in the Supporting Information shows the same event). We also confirmed the presence of fast internalization events with super-resolution live-cell STED microscopy (Figure 3f and Video S5 in the Supporting Information). Moreover, the fast internalization events were not specific to the HEK cells we used, since MDA-MB-231 cells likewise exhibited both fast desorption and internalization events (Table 2 and Figure S7 in the Supporting Information). Aside from the fast internalization, we also observed some internalization events lasting up to tens of seconds, and it appears that there are more of these events for the 100 compared to the 40 nm particles, consistent with the longer time scale experiments (Figure 2).

One potential mechanism underlying the fast internalization is that the particles are internalized via direct permeation through the plasma membrane. However, particle internalization was completely suppressed at 4 °C (Figure S8 in the Supporting Information) consistent with the particles being taken up by endocytosis, as indeed previously reported.^53^ Moreover, we can exclude that the fast internalization is due to cell membrane damage from the imaging or particle exposure, since cell integrity (Figure S3 in the Supporting Information) as well as particle uptake rate (Figure S4 in the Supporting Information) are unaffected, and fast internalization events are observed throughout the experiment time (Figure S5 in the Supporting Information). Furthermore, we performed studies with pharmacological inhibitors of several typical nanoparticle uptake pathways.^28,63−66^ Particle uptake was reduced for all particle sizes when actin polymerization was inhibited, and inhibition of macropinocytosis decreased the uptake of the 200 and 100 nm particles (Figure S9 in the Supporting Information). Moreover, it appears that cholesterol-dependent mechanisms may also contribute to particle uptake but only to a minor extent (Figure S9 in the Supporting Information). Therefore, we would anticipate internalization times comparable to those known for uptake via endocytic pathways. In general, most such endocytic routes require 30 s to several minutes to internalize larger cargo;^47−49^ in contrast, internalization takes only 1–10 s for fast endophilin-mediated endocytosis (FEME).^67^ However, to the best of our knowledge, currently there are no reports unequivocally showing nanoparticle uptake via FEME.^68^ Moreover, it has previously been shown that FEME is essentially inactive in HEK cells.^69^ It therefore seems unlikely that the rapid internalization we observe is FEME. Instead, it could be that the particles enter through an uncharacterized fast endocytic route. Alternatively, these internalization events may be particles entering through sites on the membrane where an ongoing endocytic event is already taking place, as opposed to the particles triggering the internalization event themselves.

Comparing the number of desorption to internalization events, we observed many more desorption events compared to internalization events (Figure 3a,d and Figure 3b,e). The situation is actually far more skewed in the direction of desorption, given that we are most likely missing a multitude of short-lived adsorption/desorption events at subsecond time scales,^36^ due to our temporal resolution. We can therefore conclude that the majority of particle-cell binding events are short in duration and do not lead to successful particle internalization. This is consistent with the observation of rapid internalization, as rapid desorption will bias the observations of internalization in such a way that only the rapidly internalizing particles are observed simply because the ones that would have been internalized slower desorb instead. In other words, the internalization times would be limited by the time scales of desorption processes.

### Particle Dynamics at the Cell Membrane and within the Cell

**Figure 4:**
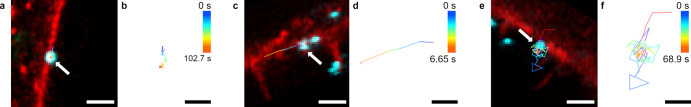
Particle dynamics at the outer cell membrane. (a, c, and e) Confocal microscopy images of particles interacting with the cell membrane and corresponding trajectories. The white arrows denote the particles of interest and the colored lines their trajectories (with the color indicating elapsed time). Scale bars = 2 μm. (b, d, and f), Zoomed-in versions of the trajectories (with the color indicating elapsed time). Scale bars = 1 μm. (a, b) 200 nm particle adsorbed onto a HEK cell membrane. The particle remains adsorbed for over 100 s and does not move substantially (≲ 1 μm) along the membrane. (c, d) 100 nm particle adsorbed onto a HEK cell membrane. In contrast to the previous example, this particle traverses several micrometers along the membrane within a few seconds. (e, f) 100 nm particle interacting with an MDA-MB-231 cell. The particle repeatedly adsorbs and desorbs from the cell membrane before finally adsorbing for 6.36 s, followed by being internalized into the cell.

In addition to the desorption and internalization times, we also observed the motion of particles at the cell membrane and immediately after internalization. After adsorption, we observed three classes of behavior: particles that adsorbed to the membrane and remained relatively stationary, particles that adsorbed and then moved along the membrane, and particles that repeatedly adsorbed on and off within a small region of the membrane (< 1 μm) (Figure 4). All three forms of dynamics were observed regardless of whether the particle ultimately desorbed from the cell or whether it was internalized. Previous studies have also reported both confined motion,^31,40,45,46^ as well as particles that explore the membrane prior to particle internalization.^30,31,45,46,70^

**Figure 5:**
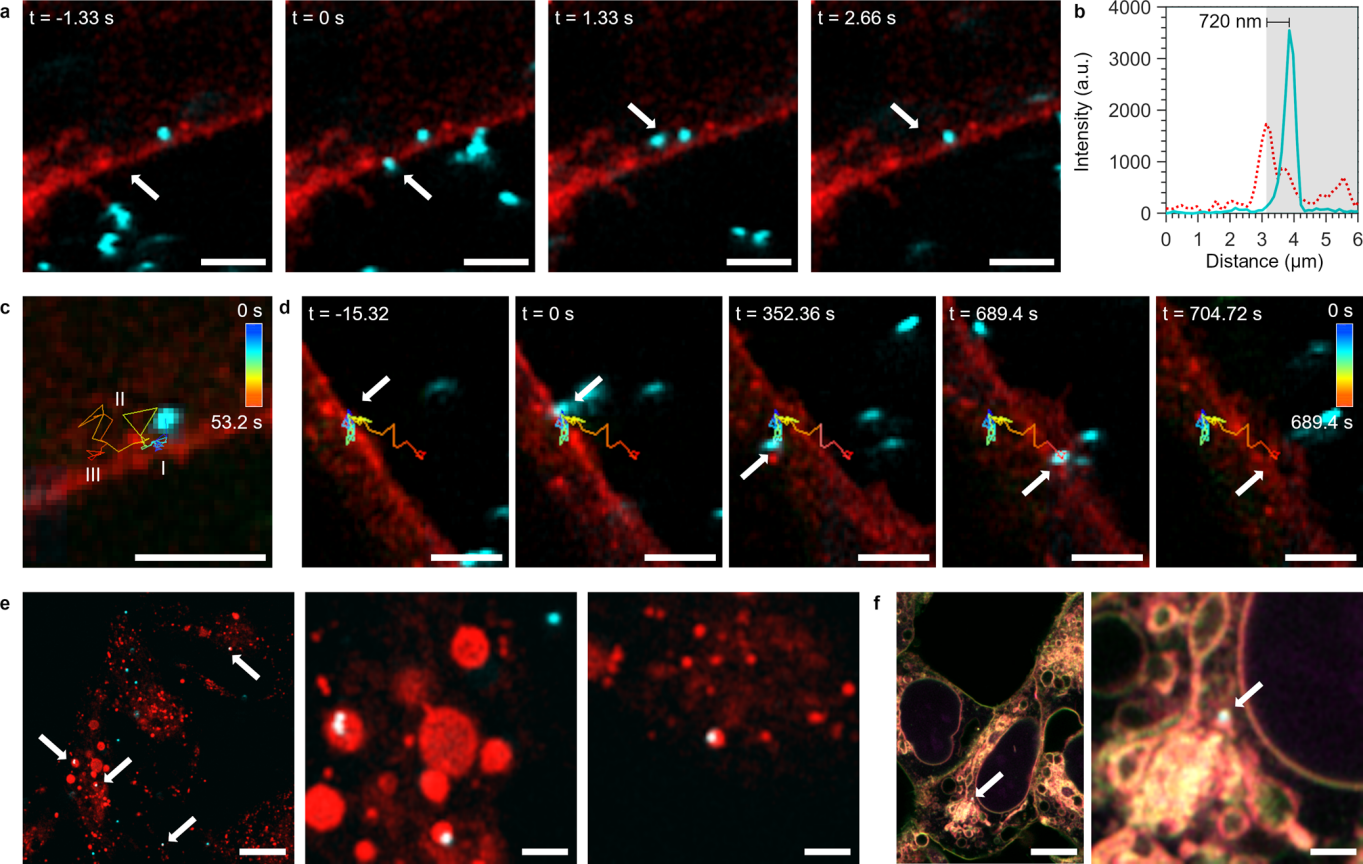
Particle intracellular dynamics. (a) Time-lapse confocal microscopy images of a particle (indicated by white arrows), which is internalized into a cell and subsequently disappears immediately after internalization. This event is also shown in Video S6 in the Supporting Information. Initially no particle is bound to the cell membrane (*t* = −1.33 s). At *t* = 0 s, the particle first appears adsorbed to the cell membrane; in the following time point (*t* = 1.33 s), the particle has been internalized, and in the next time point (*t* = 2.66 s) has disappeared. The disappearance could be due to rapid intracellular transport, motion in the axial direction or cell exit. (b) Line intensity profile of the same particle when it is within the cell (*t* = 1.33 s). The membrane signal is given by the red dotted line, whereas the nanoparticle signal is given by the cyan solid line, showing a particle–membrane separation of 720 nm. This is much larger than reported sizes for pits produced by mechanisms such clathrin or caveolin-mediated endocytosis.^41−43^ Therefore, it seems likely that the particle truly was internalized before its disappearance, as opposed to residing in a pit that was aborted and the particle then desorbing. (c) Confocal microscopy image of an internalized particle and the corresponding trajectory. The colored line denotes the trajectory (with the color indicating elapsed time). The particle remains relatively stationary in the vicinity of the outer cell membrane post-internalization (phase I of the trajectory). However, at a later stage (phase II), the particle is briefly transported further into the cell at velocities of ∼1 μm/s. The particle then returns close to the membrane and once again does not move substantially (phase III). (d) Time-lapse confocal microscopy images and corresponding trajectory of a particle being internalized into a cell and subsequently seemingly exiting. The particle of interest is indicated by the white arrows, whereas the colored line denotes its trajectory (with the color indicating elapsed time). Initially, no particle is found on the membrane (*t* = −15.32 s). The particle adsorbs in the following time point (*t* = 0 s). After residing at the membrane for some time, the particle is internalized into the cell and transported further into the cell, as clearly shown at *t* = 352.36 s. Subsequently, the particle returns toward the outer cell membrane, as exemplified at *t* = 689.4 s. In the following time point, however, the particle can no longer be visualized. We interpret this disappearance as the particle exiting the cell. Note that the cell membrane is moving and its position consequently varies across the different snapshots. (a–d) Scale bar = 3 μm. (e) Confocal microscopy images of cells exposed to 100 nm nanoparticles (cyan) for 30 min followed by a 4 h incubation to allow intracellular trafficking to occur. Lysosomes were stained with LysoTracker (red). Though some particles colocalize with lysosomes, yielding a white color in the overlay (examples indicated with white arrows in the left panel), many particles did not. Left panel shows an overview confocal image of several cells. Scale bar = 10 μm. Center and right panels show zoom-ins of particles colocalized with lysosomes. Scale bar = 2 μm. (f) Confocal microscopy images of cells incubated with 100 nm blue nanoparticles (cyan) for 30 min and stained with Nile red (red, green, and magenta). Left panel shows an overview image where both the plasma membrane and internal lipid structures have been clearly stained with Nile red. The white arrow indicates an internalized particle. Scale bar = 10 μm. The right panel shows a zoom-in of the indicated internalized particle which clearly colocalizes with the Nile red staining, yielding a white color at the nanoparticle position in the overlay. Therefore, we suggest that this particle is enveloped by an internal membrane and is unlikely to have entered the cell via direct penetration. Scale bar = 2 μm.

Once within the cell, particles also displayed various dynamics: Many particles disappeared immediately after internalization (Figure 5a). While, in principle, it is possible that such particles were not truly internalized, the distance we chose when considering a particle as internalized is sufficiently long to preclude such misidentification (Figure 1 and Figure 5b).^47,60^ Instead, it is possible that some particles, once within the cell, move in the axial direction and into a different focal plane so that we were no longer able to observe them. Moreover, intracellular transport along microtubules can reach velocities of several micrometres per second,^71,72^ so it is plausible that we were simply not able to track internalized particles transported at such speeds (see the Materials and Methods section for tracking parameters). Exit from the cell is also a possibility, as we will shortly discuss. Conversely, we were able to follow some particles that remained within the vicinity of the plasma membrane after cellular entry (Figure 5c). Some particles (including the one shown in Figure 5c) showed small bursts of motion away from the membrane but returned back to the membrane and remained relatively stationary. For a few particles, we also observed the entire process of what appears to be particle adsorption, internalization, transport within the cell, and finally exocytosis (Figure 5d). One should interpret such events with some caution, as the particle could have moved out of the plane of focus rather than exiting. However, we did not often observe particles very close to the inner side of the cell membrane moving rapidly in the *x*–*y* plane, so it would seem unlikely that all putative exit events were particles moving rapidly in the *z*-direction. It would therefore seem that these events are genuine. Interestingly, some particles followed this entire path within just minutes or even tens of seconds of entering the cell.

Furthermore, we investigated longer-term particle fate. It is commonly reported in the literature that endocytosed nanoparticles are trafficked to the lysosomes for various particle–cell systems.^13,55,66,73,74^ We observed that 32%, 39% and 31% of internalized 200, 100, and 40 nm particles, respectively, colocalized with lysosomes 4 h post-exposure (Figure 5e, as well as Figure S10 and Table S4 in the Supporting Information). This outcome is in agreement with other studies that observed that many particles do enter the lysosomes within this time span, while a significant proportion does not.^55,73^

Finally, in both the confocal and STED experiments, we observed that some particles entered cells with an associated cell membrane stain (CellMask or Abberior Star 580-DPPE) labeled structure (Figure 1e and Figure S11 in the Supporting Information), whereas many did not (Figure 1d and Figure S11 in the Supporting Information). Nile red staining, however, indicated that the far majority of internalized particles did indeed colocalize with internal membranes (Figure 5f and Figure S12 in the Supporting Information). Therefore, we conclude that the vast majority of particles enter cells via vesicles and, consequently, that the observation of rapid internalization (Figure 3) is unrelated to the absence of an observed enveloping membrane.

## Study Limitations

Our results should be interpreted with a few limitations in mind. First, we cannot rule out that desorption and internalization are affected by light exposure to the cells during the microscopy. However, the changes would have to be rather subtle as we do not detect any indications that nanoparticle uptake is disturbed by the imaging in control experiments (Figures S3–S5 in the Supporting Information).

Second, our observations are limited by both the spatial and temporal resolutions of the microscopy techniques used. Our temporal resolution implies that we are unable to resolve events that occur more rapidly than ∼1 s (for our faster experiments) or ∼15 s (for our slower experiments). This is, in particular, a limitation for our desorption measurements, where we expect multiple sub-second interactions to take place.^36^ Similarly, for our internalization measurements, it implies that there may be internalization events more rapid than reported.

Another issue that, in principle, needs to be considered is that we observed some particles that adsorbed to the cell and subsequently traversed the membrane. While we were able to follow the particles in those cases, it is possible that some particles traversed the membrane at such high speeds that we were unable to track them. Such particles will thus appear as multiple adsorption events rather than a single adsorbing event. However, previous work addressing the uptake of the same particles at a higher temporal resolution (30 ms) showed that particles on the cell membrane moved maximally a few microns across timespans up to 150 s.^31^ Consequently, we consider the likelihood of misidentifying high motility particles for multiple adsorption events to be low and, overall, do not expect this to affect the desorption and internalization time distributions that we report.

With regard to the internalization, we considered a particle to be inside only when it was 2 pixels into the cell to prevent false counts of internalization events. This rather conservative demarcation leads to a somewhat overestimated internalization time.

Overall, we thus expect that the rapid internalization we report is, if anything, slower than the real one. Nevertheless, it remains to identify the specific mechanism. We argue that direct membrane penetration can be ruled out (Figures S8 and S12 in the Supporting Information). We, furthermore, give evidence for several internalization mechanisms being at play with none being the predominant actor (Figure S9 in the Supporting Information). Therefore, we suggest the more general idea that particles enter via an already initiated endocytic event, which would be less dependent on the detailed biological mechanism.

## Conclusions

Through a combination of conventional (diffraction-limited) and super-resolution STED microscopy, we studied how long 40, 100, and 200 nm carboxylated polystyrene nanoparticles remain at the cell membrane after adsorbing to it. The majority of particles were observed to desorb from the membrane rather than being internalized, and they did so within seconds. This is unsurprising as we expect rapid dynamics at the membrane.^36^ More unexpected was the observation that, despite the particles being internalized into the cell via endocytosis,^53^ we observe that the majority of particles that do enter, do so substantially more rapidly (1–2 s) than time scales typical of endocytosis of larger cargo (e.g., 30 s to minutes for clathrin-mediated endocytosis and phagocytosis). Furthermore, this observation is independent of particle size, despite the overall uptake efficiency of these particles being size-dependent.^18^ We interpret the rapidly internalizing particles as particles that enter via an endocytic event that is already taking place rather than triggering their own internalization. For clathrin-mediated endocytosis specifically, such a mechanism has been described previously, both for viruses^45,70^ and nanoparticles.^31^ However, for nanoparticles, it was reported that only a minor fraction enters in this fashion,^31^ while we now observe that the majority of particles are taken up rapidly. In addition, we should not rule out the possibility of internalization through some as yet unknown fast endocytic route.

Next to this, particles were also observed to seemingly exit the cell, sometimes within just tens of seconds after being internalized. This suggests that cellular sorting mechanisms can operate quite rapidly. Moreover, it implies that there may be an entire population of internalized particles that are not identified by methods with long incubation periods and washing steps, as these particles may exit the cell before the measurement occurs.^75^ Whether these quickly sorted particles are relevant from a drug delivery stand point, however, remains to be resolved.

While the majority of particles desorb or are internalized rapidly (within seconds), we also observe particles that take far longer to desorb or be internalized (tens of seconds to several minutes). We may expect that receptor binding, in particular, a biomolecule in the particle corona binding specifically to its cell binding partner, leads to strong binding and, consequently, long adsorption times. The longer events we observe may hence be particles binding to a receptor and subsequently either desorbing or being internalized. In this context, it is interesting to note that while we did not observe any particle size dependence for the rapidly desorbing/internalizing particles, there may be more subtle differences between particles of different size when it comes to the longer time scale events. Indeed it has been shown that much larger microspheres are internalized on the tens of minutes time scale,^33,34^ compared to the minutes time scales for nanoparticles.^31,40^ Therefore, it seems reasonable that particle size may play a role for longer time scale events. Nevertheless, in our study, these events are clearly in the minority, and the vast majority of particles appear to be internalized more rapidly.

## Materials and Methods

### Cell Culture

Human embryonic kidney (HEK) cells (American type culture collection, no. CRL-1573, Lot No. 63966486) and MDA-MB-231 cells (LGC Promochem) were cultured in complete medium consisting of Dulbecco’s minimal essential medium (Gibco) supplemented with 10% fetal bovine serum (Gibco) at 37 °C under a 5% CO_2_ and humidified atmosphere. Regular mycoplasma tests were carried out and experiments reported are from cultures that tested negative.

### Nanoparticles

Fluorescent carboxylated polystyrene nanoparticles (“FluoSpheres”) of nominally 200, 100, and 40 nm diameter yellow-green (505/515 nm excitation/emission), 40 nm diameter dark red (660/680 nm excitation/emission), and 100 nm blue (350/440 nm excitation/emission) were purchased from Invitrogen. Particles were dispersed in complete medium at a concentration of 7.5 μg/mL, regardless of size and left at 37 °C for at least 1 h prior to usage to produce biomolecular corona-covered particles.

Particle dispersions were characterized by dynamic light scattering and laser Doppler velocimetry using a Malvern ZetaSizer Nano ZS (Malvern Instruments) and ZetaSizer Software version 7.13 (Malvern Instruments). Dispersions were prepared as above but at a concentration of 50 μg/mL and were compared to pristine samples in which phosphate buffered saline (Gibco) was used as a dispersant rather than complete medium. The reported results are the mean and standard deviation of three repeat measurements with minimally 10 runs each.

### Confocal Microscopy

#### Particle-Cell Membrane Interaction Experiments

35 mm Petri dishes with glass bottom microwells (No. 1.5, MatTek Corp) were seeded with HEK cells 2 days before the experiments. The cell membrane stain was prepared by adding CellMask Orange Plasma Membrane Stain (Invitrogen) to complete medium for a concentration of 2.5 μg/mL after which it was heated to 37 °C. Prior to imaging, cells were incubated with the cell membrane stain for 5 min at 37 °C. Subsequently, the cell membrane stain was aspirated and replaced with 1 mL of 37 °C biomolecular corona-covered particle dispersion and placed on the microscope which was preheated to 37 °C with 5% CO_2_.

Confocal images were taken using a CellDiscoverer 7 (Zeiss) with an LSM900 confocal head with the AiryScan 2 detector, and a 50× plan apochromatic water immersion objective (with autocorrection rings). The Definite Focus setting was used to maintain focus during the image acquisition. The 488 nm excitation laser with a 490/575 nm filter was used to image the yellow/green nanoparticles, while CellMask Orange was imaged using a 561 nm excitation laser and a 565/700 nm filter. All images were processed by using the Airyscan Processing step in the microscope operating software (ZEN blue 3.5, Zeiss). The Airyscan detector and processing achieves an *xy* resolution of 120 nm and a *z* resolution of 350 nm.^76^ Entire cells or multiple cells were imaged in a single field of view (58.78 μm × 58.78 μm).

Longer time scale (15 s) experiments were performed in three dimensions, first imaging the membrane channel and then the particle channel, before moving to the next focal plane. Three focal planes were imaged in this way before returning to the initial focal plane and repeating. The interval between successive time points (z stacks) was ∼15 s and a total of 40 time points were measured. Shorter time scale (1.5 s) experiments were performed in two dimensions. In this case, the membrane channel was first imaged once, after which only the nanoparticle channel was successively imaged at an ∼1 s rate for 80 frames, followed by a second imaging of the membrane channel. The two membrane images were superimposed and line profiles were used to identify regions where the cell membrane overlapped (i.e., where the membrane had not visibly moved while the particle channel was being imaged) and only these regions were analyzed.

To estimate the particle localization error, data were collected for nanoparticles adsorbed on glass (in the absence of cells) imaged using the same settings as the shorter time scale experiments.

#### Cell Integrity Control Experiment

For the cell integrity control, Sytox orange (Thermo Fisher Scientific) was dispersed in complete medium at a 125 nM concentration and heated to 37 °C. Cells in the presence of 40 nm nanoparticles were exposed to the imaging conditions of either the longer time scale or shorter time scale confocal microscopy experiments (see above), after which the particle dispersion was removed and cells were incubated with Sytox dispersion. As a negative control, healthy cells that had not been exposed to nanoparticles nor laser illumination were used, whereas as a positive control, cells incubated with 70% ethanol before Sytox incubation were used. Assessment of whether the cell nuclei were stained with Sytox was performed using the wide-field fluorescence modality. 50× and 20× plan apochromatic objectives were used with a 590 nm LED and 545/630 nm filter.

#### Cell Nanoparticle Uptake Control Experiment

Cells were exposed to 40 nm nanoparticles, after which the cells were placed on the microscope and imaged according to the conditions of either the longer time scale or shorter time scale confocal microscopy experiments (see above). Control cells were likewise exposed to 40 nm nanoparticles for the same timespan as the imaged cells, but they were not exposed to laser irradiation but instead kept in an incubator at 37 °C with 5% CO_2_. Both samples were washed with Live Cell Imaging Solution (Invitrogen) before confocal images were taken using a 5× plan apochromatic objective. The signal in the nanoparticle channel was used as an indication of particle uptake. Cells were manually segmented and the total nanoparticle signal intensity for individual cells in the images was measured using ImageJ/Fiji.^77,78^ This was compared for the irradiated and nonirradiated cells.

#### Cell Energy Depletion Experiments

For the cell energy depletion control experiment, cells were first stained with the cell membrane stain at 37 °C and subsequently kept at 4 °C for 30 min. This was followed by exposure to nanoparticles for 1 h at 4 °C before washing and finally fixation with paraformaldehyde (4%; VWR). To compare to the energy-depleted cells, other cell samples were subjected to the same procedure but were maintained at 37 °C throughout.

#### Particle-Lysosome Colocalization Experiments

Cells were incubated with 40, 100, or 200 nm yellow/green nanoparticle dispersion for 30 min followed by washing and further incubation for 4 h at 37 °C. Cells were stained with LysoTracker Red (Invitrogen) dispersed in phosphate buffer saline at a concentration of 0.75 μM for 1 h. The LysoTracker dispersion was washed away and replaced with Live Cell Imaging Solution. Snapshots of cells were then taken using a CellDiscoverer 7 microscope with the same settings as the particle-cell membrane interaction experiments (see above), where the green channel was used to visualize the particles and the orange/red channel LysoTracker.

#### Particle-Internal Membrane Colocalization Experiments

Cells were incubated with a 100 nm blue nanoparticle dispersion for 30 min, followed by washing and fixation with 4% paraformaldehyde. Nile red (Sigma-Aldrich) was dispersed in DMSO to a concentration of 10 mM and then further diluted in phosphate buffer saline to a concentration of 1 μg/mL. Nile red dispersion was added to the fixed cells and remained there during imaging. Confocal imaging was performed with a CellDiscoverer 7 microscope using the Airyscan detector and 50× objective. The 405 nm excitation laser and 400/490 emission was used to visualize the nanoparticles, whereas the broad Nile Red spectrum was imaged using the yellow/green (488 nm excitation, 490/575 nm emission), orange/red (561 nm excitation, 565/700 nm emission), and dark red (640 nm excitation, 650/700 nm emission) channels.

### STimulated Emission Depletion (STED) Microscopy

HEK cells were seeded onto borosilicate #1.5 18 mm coverslips (Marienfeld, 0117580, Lot 43862-831) one or 2 days prior to experiments. Abberior Star-580-DPPE (Abberior GmbH) was used as the cell membrane probe. The membrane probe stock powder was diluted in DMSO to a concentration of 1 mg/mL and further diluted in Live Cell Imaging Solution prior to experiments to achieve a final concentration of 1 ng/mL. The cell membrane stain was briefly vortexed, then sonicated in a bath sonicator for 10 min, and finally heated to 37 °C. Prior to imaging, the cells were washed twice with Live Cell Imaging Solution and then incubated with the cell membrane stain for 10 min at 37 °C, after which the cells were washed again and 40 nm dark red nanoparticle dispersion was added. The slides were transferred to live-cell imaging chambers (Live Cell Instrument, CM-B18-1, magnetic imaging chamber for 18 mm coverslips), which was placed on an Abberior Expert Line microscope (Abberior Instruments GmbH) which was preheated to 37 °C with 5% CO_2_. Two-color live-cell STED images were taken using a 100× oil immersion objective (Olympus Objective UPlanSApo 100×/1.40 Oil) and 37 °C refractive index matching oil (Cargille Laboratories, Type 37LDF). 561 nm (26–46 μW at laser head) and 640 nm (24–60 μW at laser head) lasers were used to excite the membrane and nanoparticles, respectively, and a 775 nm STED laser (90–180 mW at laser head) was used. The pinhole was set to 1.0 AU. The emission was collected using avalanche photodetectors with a spectral range of 605 ± 30 and 700 ± 50 nm for the membrane and nanoparticles, respectively. Small segments of the outer cell membrane were imaged to achieve sufficient temporal resolution (∼0.5–7 s per frame) with a 45 nm pixel size, a pixel dwell time of 10 μs, and two line steps for both channels. Overall, small segments from over 25 different cells were imaged. From this data, we captured 11 internalization events from 9 different cells. Further details about the STED settings for the individual datasets shown in Figures 1 and 3 are given in the section entitled “STED Imaging Parameters” in the Supporting Information.

### Flow Cytometry

#### Uptake Kinetics

Cells were exposed to 40, 100, and 200 nm nanoparticles at the same concentration as used across the previous experiments (7.5 μg/mL). The particles were incubated with cells for various timespans ranging from 0 to 60 min, followed by sample preparation, flow cytometry measurement, and analysis as described below. Data are reported in terms of the mean cell fluorescence of the total sample and the standard error of the mean.

#### Inhibitor Studies

Chlorpromazine hydrochloride (Sigma–Aldrich), 5-(*N*-ethyl-*N*-isopropyl)amiloride (EIPA, Sigma–-Aldrich), cytochalasin D (ThermoFisher Scientific) and methyl-β-cyclodextrin (MβCD, Sigma–Aldrich) were used as inhibitors at concentrations of 10 μg/mL, 50 μM, 2.5 μg/mL and 2.5 mg/mL, respectively.^28^ Pre-exposure dispersions were prepared by adding the compounds to complete medium or serum-free medium in the case of methyl-β-cyclodextrin. Nanoparticle dispersions were prepared in complete medium 1 h in advance and divided into separate tubes, and the compounds were added to each just prior to exposure. Cells were incubated with pre-exposure inhibitor dispersions for 10 min, followed by 3 h (chlorpromazine hydrochloride, EIPA and cytochalasin D) or 30 min (MβCD) incubation with the nanoparticle dispersions containing the inhibitors. Alternatively, control cells were incubated with nanoparticle dispersions for the same amount of time. Positive controls were performed using transferrin-Alexa 546 (10 μg/mL, ThermoFisher Scientific), TRITC-dextran (250 μg/mL, ThermoFisher Scientific), morphological imaging, and BODIPY FL C5-lactosylceramide/BSA complex (LacCer, 0.0125 μM, Fisher Scientific) for the chlorpromazine hydrochloride, EIPA, cytochalasin D and MβCD conditions, respectively.^28^ Cells were then harvested, measured with flow cytometry, and analyzed as described below. Experiments were performed in triplicate and are reported in terms of the mean and standard deviation across all repeats.

Significance testing was performed using rank-based methods.^79^ All replicates from the same experiment, including those of the control, were normalized to the mean value of the control (cells exposed to nanoparticles in the absence of the inhibitor). Subsequently, a one-sided Mann–Whitney test was performed using SciPy^80^ (version 1.3.3) on the normalized replicates from all independent experiments pooled together, evaluating whether the cells exposed to an inhibitor exhibited a lower particle uptake than control at a significance level of 0.05.

#### Flow Cytometry Analysis

Cells were washed once with complete medium and twice with phosphate buffered saline, followed by trypsinization (Gibco). The harvested cells were centrifuged at 250 rcf for 5 min, and the pellet was resuspended in phosphate buffered saline. Cell dispersions were then measured by using a NovoCyte Quanteon flow cytometer. Nanoparticles were excited with a 488 nm laser and measured at 530/30 nm. Cellular debris was removed using forward and side scattering areas, after which cell doublets were removed using forward scattering height and area. After these filtering steps, ∼15 000 cells were measured per sample. The data were analyzed using Kaluza Analysis software (v2.1). The average nanoparticle uptake by cells was assessed as the arithmetic mean cell intensity in the nanoparticle channel of the resultant data.

#### Microscopy Data Analysis

Image analysis was performed in ImageJ/Fiji.^77,78^ Background removal was performed on the confocal data of the 40 nm nanoparticles (due to observed accumulation of leaked dye within the cells) by blurring the nanoparticle channel of each image with a Gaussian filter of 5 pixel radius and subtracting the result from the original data. No background subtraction was performed on the 100 and 200 nm nanoparticle data.

Particles were tracked in the areas close to the outer cell membrane, as identified from the cell membrane stain. In the case of the 2D experiments, this was only performed for regions where the before and after membrane images overlapped. For the three-dimensional (3D) data, particle identification and tracking was performed manually using the ImageJ/Fiji plugin TrackMate,^81^ using the Manual tracking option. Particles were tracked across time and through all three focal planes. Automated tracking was performed for the 2D experiments using TrackMate.^81^ The built-in sub pixel localization and median filtering functions were used to identify the particles. Particles were linked to obtain trajectories by using a linking distance of 0.8, 1.4, and 2.4 μm for 40, 100, and 200 nm particles, respectively. These values were chosen as they were twice the size of the estimated object size. This criterion was used in order to minimize misidentification of particles that adsorbed and desorbed in the vicinity of each other as the same particle yet also allow for the tracking of particles that diffused on the cell membrane. No gap linking was permitted. Therefore, particles that moved at velocities greater than 0.5–2.4 μm (dependent on the trajectory linking distance used and imaging rate) would not be identified as a singular particle trajectory. Trajectories were then manually checked for correctness and to find events of interest.

Fluorescence intensity line profiles were used to determine the position of the particles with respect to the cell membrane. The membrane position was considered to be the first peak in the membrane intensity along the cross section (see Figure 1f–j for examples of membrane peak identification). From this identification, the inside of the cell was defined as the region following the membrane peak. We note that peaks in the membrane signal intensity within the cells were also observed due to internalized sections of membranes (e.g., vesicles). Particles were considered to be adsorbed when the peak in the particle intensity signal coincided with the membrane signal yet was not inside of the cell (as defined by the position of the membrane peak) (Figure 1f,h). Particles were identified as internalized when the particle position was within the cell, and the maximum intensities of the membrane and particle signal were separated by at least 2 pixels (240 nm for confocal and 90 nm for STED microscopy; see Figure 1g,i,j).

Events were then ascribed an internalization time as follows: the time between the first frame in which an object was identified as being adsorbed to the membrane and the first frame in which the object was identified as internalized. Desorption times were defined as the time between the first and last time frames in which an object was identified as adsorbed, given that the object was not internalized. Desorption events minimally contained two successive time points in which the particle was identified as adsorbed to ensure that identified events were indeed particles interacting with the cell membrane rather than simply freely diffusing in the vicinity of the membrane at the time point the image was captured. Particles still adsorbed to the cell membrane at the end of the observation time were discarded from the dataset as they could not be ascribed neither a desorption nor an internalization time. The desorption and internalization times were averaged across all events (per condition) by calculating the mean and standard error. Furthermore, the characteristic time scale, τ, was determined by fitting an exponentially decaying function to the distribution.

The localization precision of the 2D experiments was determined by tracking particles adsorbed to glass (in the absence of cells) by using the same analysis procedure as described above. The localization precision quoted herein is the root-mean-square displacement between two consecutive frames of a population of particles (*n* = 3049, 795, and 463 for 40, 100, and 200 nm particles, respectively) with its standard error of the mean as error.

## Supporting Information Available

Particle dispersion characterization; controls for imaging and nanoparticle exposure conditions; particle uptake kinetics; average desorption and internalization times; experiments on MDA-MB-231 cells; control for cell energy dependent particle uptake; nanoparticle uptake mechanism assessed with pharmacological inhibitors; nanoparticle colocalization with lysosomes; membrane envelopment of internalized particles; STED imaging parameters; supporting video captions (PDF)
Supporting video S1 (AVI)
Supporting video S2 (AVI)
Supporting video S3 (AVI)
Supporting video S4 (AVI)
Supporting video S5 (AVI)
Supporting video S6 (AVI)

## Supplementary Material

nn3c01124_si_001.pdf

nn3c01124_si_002.avi

nn3c01124_si_003.avi

nn3c01124_si_004.avi

nn3c01124_si_005.avi

nn3c01124_si_006.avi

nn3c01124_si_007.avi
