# Supplementary material for: Rapid Internalization of Nanoparticles by Human Cells at the Single Particle Level
Source: ACS Nano. 2023 Aug 29;17(17):16517–29. doi: 10.1021/acsnano.3c01124 (PMC10510712; doi:10.1021/acsnano.3c01124)
Supplement: Supplementary file 1 — nn3c01124_si_001.pdf [file nn3c01124_si_001.pdf]

Supporting Information to

Rapid internalization of nanoparticles by human cells at the single particle level

Ceri J. Richards[a,b], Thomas C. Q. Burgers[b], Rifka Vlijm[b], Wouter H. Roos[b*], and Christoffer Åberg[a*]

[a] Pharmaceutical Analysis, Groningen Research Institute of Pharmacy, University of Groningen, 9713 AV Groningen, Netherlands

[b] Molecular Biophysics, Zernike Institute for Advanced Materials, University of Groningen, 9747 AG Groningen, Netherlands

[*]Email: christoffer.aberg@rug.nl; w.h.roos@rug.nl.

Particle dispersion characterization

Supporting Table S1. Physicochemical characterisation of the particle dispersions in terms of size and ζ potential. Dynamic light scattering (DLS) was used to measure the size and laser Doppler velocimetry to measure ζ potential. The nanoparticles were dispersed in phosphate buffered saline (PBS) to characterize the pristine nanoparticles. The results show that for all the particles we used, the pristine particle diameters are close to the nominal diameters given by the manufacturer and that the particles are fairly monodisperse. Furthermore, the surface carries a negative charge, as reflected by the negative ζ potential. To characterize the dispersions applied on cells, we also dispersed the particles in cell culture medium supplemented with 10% foetal bovine serum (cDMEM). The formation of a biomolecular corona on the particles in medium is clearly seen from the shift of the ζ potential to more neutral values. We also observe an increase in the size of the particles, though we hasten to add that these numbers should not be interpreted in an absolute sense due to the fact that the underlying analysis is not ideal for a complex fluid like particles in medium. Corresponding size distributions are reported in Supporting Figure S1. All in all, the results show that uncontrolled agglomeration does not take place upon dispersion in medium, in line with previous studies on the yellow-green particles.[1–4] Further evidence that the 40 nm yellow-green particles remain monomeric in cDMEM is shown in Supporting Figure S2. Values quoted are the mean ± standard deviation of three repeat measurements.

Particle	Dispersant	*z* average diameter (nm)[1]	Polydispersity index[2]	Peak diameter (nm)[3]	ζ potential (mV)
40 nm yellow-green	PBS	55 ± 0.8	0.09 ± 0.05	59 ± 1	−32 ± 2
	cDMEM	146 ± 3	0.25 ± 0.002	192 ± 7	−10 ± 0.7
40 nm dark red	PBS	61 ± 2	0.14 ± 0.02	67 ± 3	−36 ± 4
	cDMEM	89 ± 1	0.31 ± 0.02	131 ± 4	−10 ± 2
100 nm yellow-green	PBS	108 ± 0.6	0.03 ± 0.01	113 ± 0.8	−40 ± 3
	cDMEM	146 ± 1	0.11 ± 0.02	165 ± 3	−10 ± 0.4
100 nm blue	PBS	118 ± 4	0.04 ± 0.02	125 ± 4	−32 ± 0.9
	cDMEM	147 ± 2	0.11 ± 0.01	164 ± 3	−11 ± 1
200 nm yellow-green	PBS	205 ± 0.4	0.03 ± 0.01	214 ± 2	−44 ± 1
	cDMEM	258 ± 3	0.08 ± 0.02	282 ± 4	−9 ± 0.6

1 From cumulant analysis.

2 From cumulant fitting.

3 Diameter of main peak from CONTIN analysis.

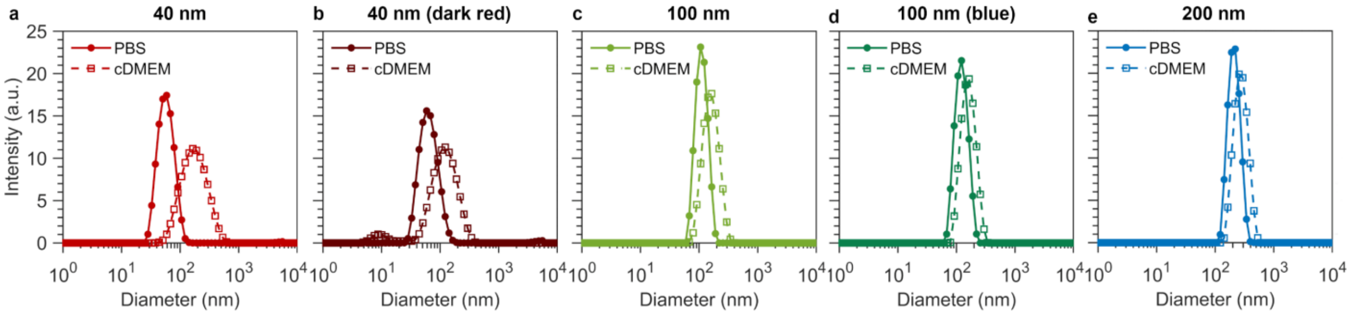

Supporting Figure S1. Nanoparticle size distributions characterized by dynamic light scattering (DLS). The different particles were dispersed in phosphate buffered saline (PBS; filled circles) to characterize the bare particles, and in cell medium with serum (cDMEM; open squares) to characterize the dispersions as exposed to the cells. **(a)** 40 nm yellow-green nanoparticles; **(b)** 40 nm dark red nanoparticles; **(c)** 100 nm yellow-green nanoparticles; **(d)** 100 nm blue nanoparticles; and **(e)** 200 nm yellow-green nanoparticles. Upon dispersion in cell medium, a biomolecular corona forms on the particle surface, resulting in an increased hydrodynamic diameter for all particles; it should be noted, though, that the magnitude of the shift should not be interpreted in absolute terms due to the complexity of the medium. In this context, we also note that for the 40 nm dark red particles dispersed in medium (panel b, dotted line) there is a minor population at ~10 nm. We interpret this to be biomolecular agglomerates in the medium, something which is often (but not always) observed for particles of this size[5,6] (for larger particles the scattering of the particles completely dominates over any potential smaller agglomerates and consequently the agglomerates are not observed). Corresponding mean and peak diameters are reported in Supporting Table S1.

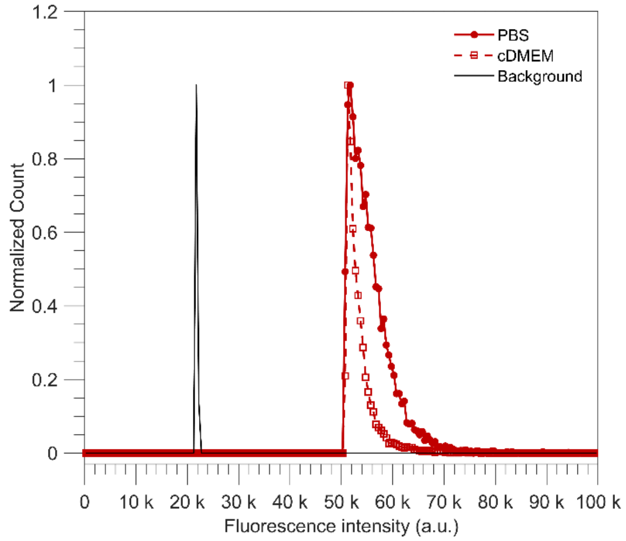

Supporting Figure S2. Further characterization of the size of the 40 nm yellow-green nanoparticles using fluorescence. To show that the 40 nm yellow-green nanoparticles, whose diameter in cDMEM characterized by DLS is quite big (Supporting Table S1), do not agglomerate in complete medium, we also characterized their fluorescence distribution by confocal microscopy. As a control, the particles were dispersed in phosphate buffered saline (PBS) at a concentration of 0.075 µg/ml and the dispersion was incubated on glass slides for 10 min, after which the slides were washed to remove unbound particles. Confocal images were taken of the slides and the total fluorescence intensity of each identified particle was recorded (filled circles). This procedure was repeated for the particles dispersed in cDMEM (open squares) at the increased concentration of 7.5 µg/ml due to the reduced binding of biomolecular corona-covered particles to the glass compared to pristine particles. Likewise, the background signal was characterized (black solid line). The distributions were normalized to the peak maximum to compare the conditions. We observe that the pristine particles (dispersed in PBS) exhibit a single peak at a fluorescence of 55500 ± 4200 (mean ± standard deviation). The DLS results above (Supporting Table S1) shows that the 40 nm yellow-green particles do not agglomerate in PBS so we can conclude that this single peak corresponds to single particles. We would expect that dimers would exhibit a fluorescence signal two-fold that of a single particle, as we have indeed observed previously for the 100 nm yellow-green particles.[7] Accounting for the background signal of around 22,000 then suggests that a dimer would have a fluorescence of around 85,000–90,000. Larger agglomerates would obviously exhibit an even larger fluorescence. However, for the particles dispersed in cDMEM, we observe only a single peak at a fluorescence of 53600 ± 3600 (mean ± standard deviation) very similar to that of the bare particles. The fact that the peaks coincide for pristine particles and biomolecular corona-covered particles, coupled to the fact that no additional peaks corresponding to dimers or agglomerates are observed, shows that the particles dispersed in cDMEM do not agglomerate.

Controls for imaging and nanoparticle exposure conditions

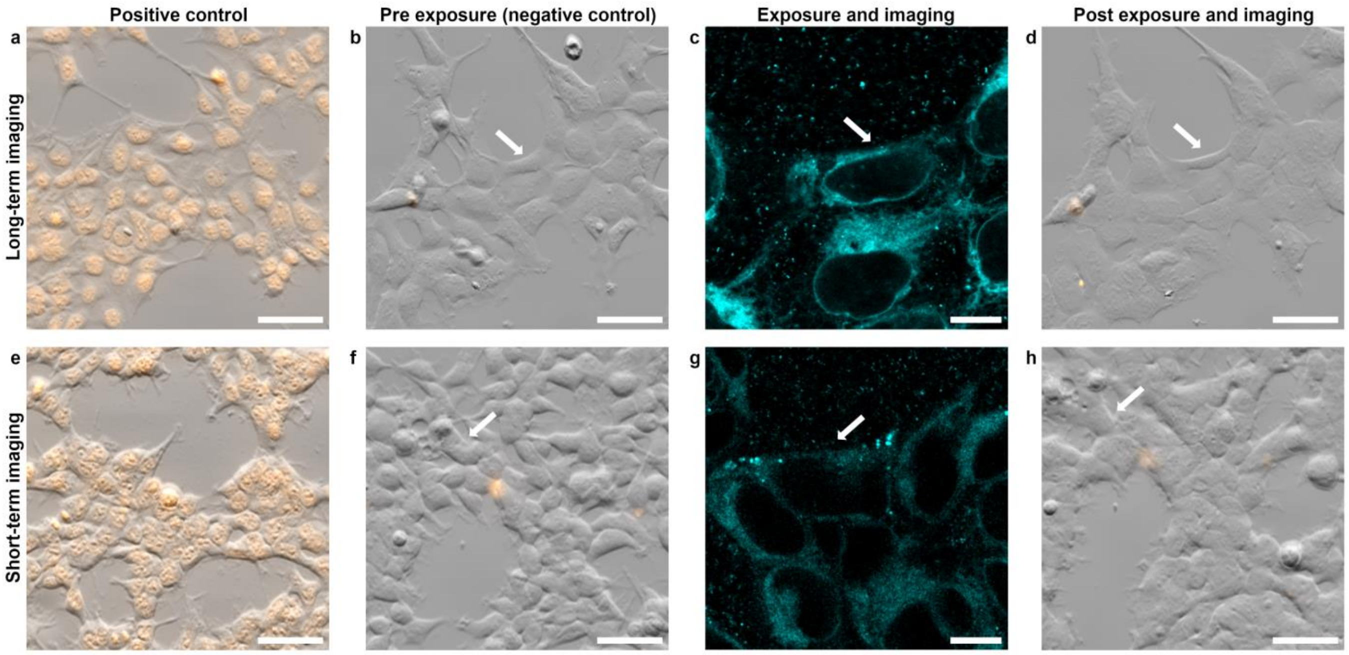

Supporting Figure S3. Nanoparticle exposure and imaging do not affect cellular integrity of HEK cells. To assess whether the particle exposure and the imaging conditions applied in this work affected cell integrity, cells were exposed to Sytox orange. This dye permeates into cells and stains the nucleus if the outer cell membrane is compromised. **(a–b, d–f, h)** Phase gradient contrast (grey) and widefield fluorescence images of cells incubated with Sytox (yellow). Scalebars 50 µm. **(a, e)** Positive controls. Cells were exposed to 70% ethanol for 10 min prior to Sytox staining. Sytox staining of the nuclei is clearly visible, indicating that Sytox does indeed stain compromised cells. **(b–d, f–h)** Cells subjected to Sytox staining both before and after nanoparticle exposure and **(d)** long-term imaging or **(h)** short-term imaging. Note that the same cells were investigated and white arrows points to the same cell in all images. **(b, f)** Negative controls. Cells were incubated with Sytox under pre-experimental conditions (*i.e.*, no nanoparticle exposure nor imaging). Under these conditions, cells show no Sytox staining. **(c, g)** Confocal microscopy images of the same cells at enlarged magnification. These cells were exposed to 40 nm yellow-green nanoparticles (cyan) and subjected to the same **(c)** long-term imaging or **(g)** short-term imaging as applied for the main results of this work (see Experimental section 'Particle-cell membrane interaction experiments' for details). Scale bar 10 µm. **(d, h)** After exposure, the cells were re-incubated with Sytox and imaged. Compared to before exposure (panel b and f), cells displayed some changes in morphology after imaging. Note, though, that we would not have included events taking place at membranes that showed major disruption or blebbing. Importantly, cells did not show positive Sytox staining, showing that the combination of nanoparticle exposure and imaging did not compromise cell integrity.

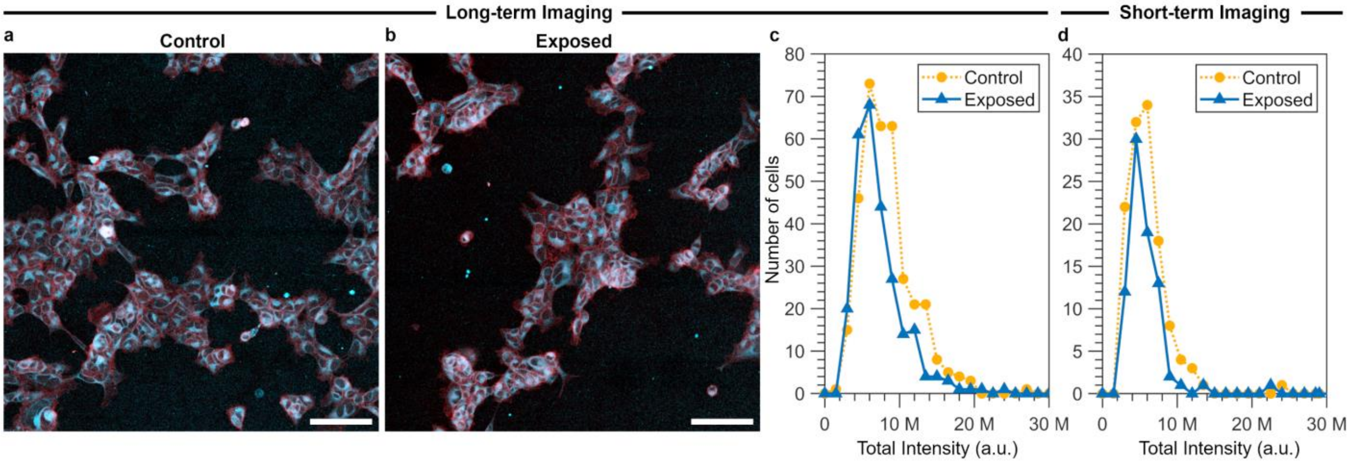

Supporting Figure S4. Particle uptake is not affected by imaging conditions. We aimed to assess whether the imaging conditions used in this work affected cellular functioning, specifically, nanoparticle uptake efficiency. That is, was uptake reduced or increased due to the imaging? To this end, we compared populations of HEK cells that were incubated with 40 nm yellow-green nanoparticles for the same period of time: one population was not subjected to imaging (control conditions), whereas the other was subjected to the same long-term or short-term imaging as applied for the main results of this work (see Experimental section 'Particle-cell membrane interaction experiments' for details). Afterwards, cells were washed to remove unbound particles and low-magnification images were acquired. Cells were manually identified in the images and the total nanoparticle fluorescence intensity measured for each cell. These values were used as an indication of nanoparticle uptake and were compared for control and imaged cells to assess any notable differences. **(a–b)** Example confocal microscopy images of populations of cells stained with CellMask Orange (red signal) and incubated with 40 nm yellow-green nanoparticles (cyan). Scalebars 100 µm. **(a)** Control conditions. **(b)** Cells subjected to long-term imaging. **(c)** Comparison of the total nanoparticle fluorescence intensity per cell under control conditions and for cells subjected to long-term imaging. **(d)** Comparison of the total nanoparticle fluorescence intensity per cell under control conditions and for cells subjected to short-term imaging. In both panel c and d, the distribution of fluorescence intensity is similar for cells that have not been imaged and cells that have, indicating that neither long-term nor short-term imaging notably promote or inhibit nanoparticle uptake.

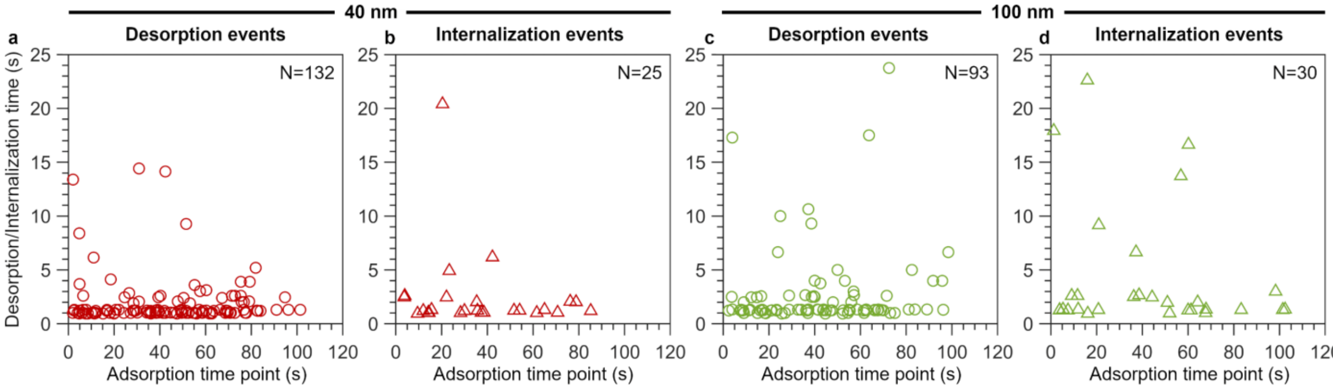

Supporting Figure S5. Rapid nanoparticle-cell internalization and desorption events are not promoted due to imaging conditions. Desorption or internalization time *vs.* the time during imaging when a particle adsorbed to the cell membrane for the events displayed in the histograms of Figure 3 (short-term imaging conditions). **(a)** Desorption events and **(b)** internalization events for cells exposed to 40 nm particles. **(c)** Desorption events and **(d)** internalization events for cells exposed to 100 nm particles. For both particle sizes, rapid internalization events occur during the early stages, as well as the late stages of imaging. The same observation is found for the desorption events. Therefore, we conclude that rapid internalization is not artificially induced by ongoing imaging. We note that particles still adsorbed to the membrane at the end of the observation time were discarded from the data set; therefore the recorded desorption/internalization time is limited by the total imaging time, *i.e.*, particles included in our data set that adsorbed within the last 10 s of observation cannot have a desorption/internalization time greater than 10 s.

Particle uptake kinetics

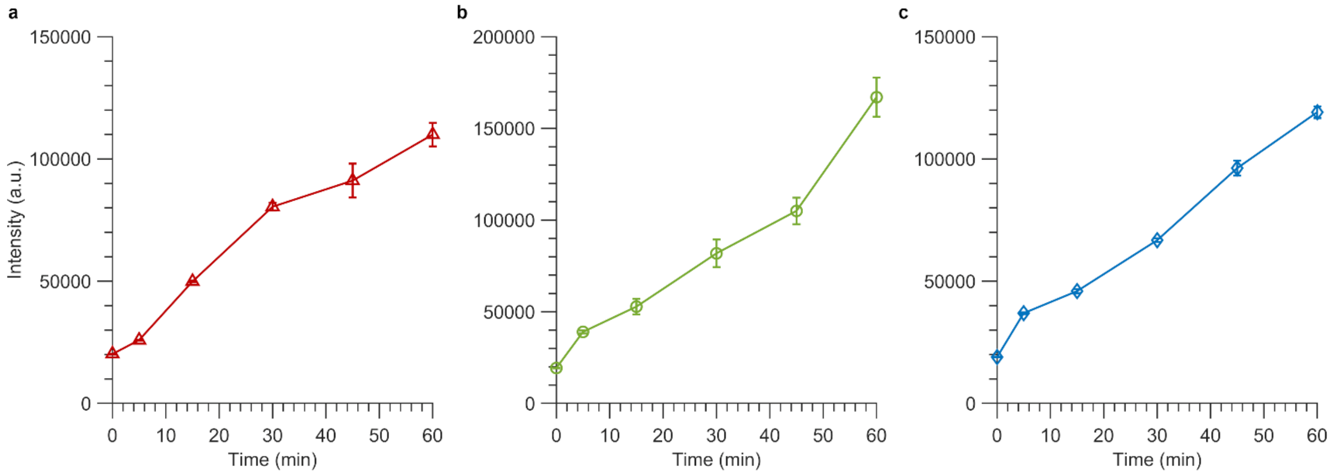

Supporting Figure S6. Particle uptake kinetics. HEK cells were exposed to dispersions of the 40, 100 and 200 nm yellow-green nanoparticles for various timespans ranging from 0 to 60 min. The same concentrations were used as for the results presented in the main text (Figure 2–3). Cell fluorescence was then measured by flow cytometry to assess particle uptake. Average cell intensity as a function of exposure time for **(a)** 40 nm particles; **(b)** 100 nm particles; and **(c)** 200 nm particles. We note that the intensity is an indication of the *total* number of particles associated with a cell, *i.e.,* both internalized particles and those strongly adsorbed to the cell membrane.[1] For all particles sizes, we observe an initial increase in average cell fluorescence within the first few minutes of exposure, indicative of particle adsorption to the cells. After this initial increase, all particles sizes show a steady increase in cell fluorescence across time, showing that there is no uptake saturation mechanisms present for the timespans or particle concentrations used within our experiments. Error bars represent standard error of the mean over cells.

Average desorption and internalization times

Supporting Table S2. Typical desorption and internalization times for the 3D experiments. We used two different measures to characterise the desorption and internalization times. First, we simply used the arithmetic mean, which is reported here together with its associated standard error. Second, since the arithmetic mean is sensitive to outliers, we also performed an exponential fit of the equation $N(t) = N_0 e^{-t/\tau}$, where τ is the characteristic time, to the data. The characteristic times are also reported in Table 1. All fits had an R^2 value of 0.95 or more. In general, the mean times are larger than the times extracted by fitting, consistent with the mean being influenced by the distribution tails more. For desorption, both quantities show the same behaviour that the typical desorption time is largely independent of particle size. For internalization there is some discrepancy between 100 nm particles and the other two particle sizes in terms of the mean time, which is substantially higher for the 100 nm particles. The source of this discrepancy may be found in Figure 2d–f, where it is clear that the 100 nm particles exhibit more long-lived internalization events.

Particle	Mean desorption time (s)	Characteristic desorption time (s)	Mean internalization time (s)	Characteristic internalization time (s)
40 nm	30 ± 2	16	38 ± 7	20
100 nm	28 ± 2	12	69 ± 19	13
200 nm	21 ± 3	19	25 ± 9	12

Supporting Table S3. Typical desorption and internalization times for the 2D experiments. We used two different measures to characterise the desorption and internalization times. First, we simply used the arithmetic mean, which is reported here together with its associated standard error. Second, since the arithmetic mean is sensitive to outliers, we also performed an exponential fit of the equation $N(t) = N_0 e^{-t/\tau}$, where τ is the characteristic time, to the data. The characteristic times are also reported in Table 2. All fits had an R^2 value of 0.95 or more. In general, the mean times are larger than the times extracted by fitting, consistent with the mean being influenced by the distribution tails more. For desorption and internalization, both quantities show the same behaviour that the typical desorption time is largely independent of particle size.

Particle	Mean desorption time (s)	Characteristic desorption time (s)	Mean internalization time (s)	Characteristic internalization time (s)
**HEK cells**				
40 nm	1.9 ± 0.2	1	2.5 ± 0.8	1
100 nm	2.6 ± 0.4	1	4.2 ± 0.1	2
200 nm	4.8 ± 2.0	2	-	-
**MDA-MB-231 cells**				
100 nm	1.7 ± 0.2	0.7	2.0 ± 0.4	1.3

Experiments on MDA-MB-231 cells

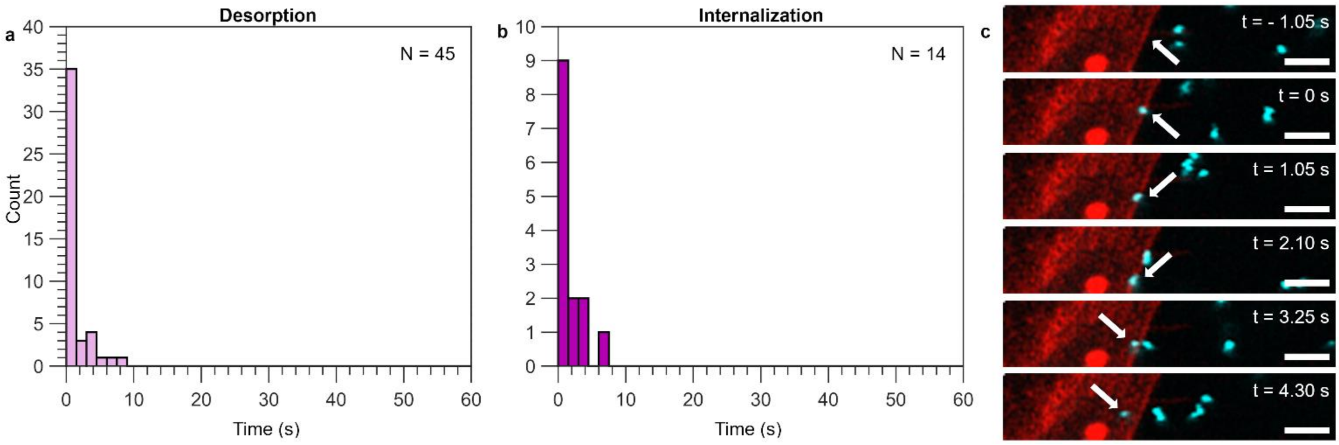

Supporting Figure S7. Particle desorption and internalization times for 100 nm particles exposed to MDA-MB-231 cells. To check whether the fast internalization events observed for HEK cells were not due to a cell-line-specific mechanism, we also investigated the interaction of nanoparticles with the epithelial breast cancer cell line MDA-MB-231. Cells were incubated with 100 nm nanoparticles and observed at a temporal resolution of ~1.5 s (*cf.* the results in Figure 3 for HEK cells). Histograms of particle **(a)** desorption times and **(b)** internalization times. As with the HEK cells (Figure 3) the majority of particles were adsorbed for less than 5 s and therefore the fast internalization events do not appear to be specific to the HEK cells. The number of events the data is based on is indicated in the graphs. Histogram bin sizes 1.5 s. Results from 20 cells. **(c)** Confocal microscopy images of a particle (cyan) being internalized by an MDA-MB-231 cell (particle indicated by the white arrows). At t = −1.05 s, no particle is observed. The particle is first visualized adsorbed onto the cell membrane (red) at t = 0 s. In the following time frames it remains adsorbed and moves small distances along the membrane, after which the particle is internalized into the cell at t = 4.30 s. Scale bars 3 μm.

Control for cell energy dependent particle uptake

a b c

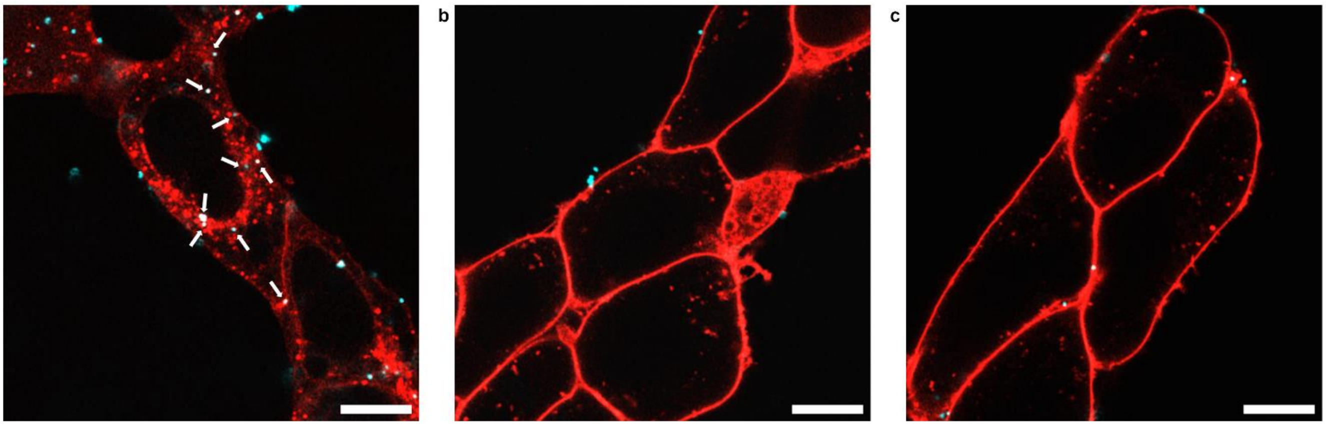

Supporting Figure S8. Particle internalization is suppressed at 4 °C. In general, the particles used here are internalized by cells by endocytosis, which is cell energy-dependent,[8] but to demonstrate that this is true also for the HEK cells, we aimed to show it using the same set up as for the rest of this work. Thus, we compared particle uptake by cells subjected to a prior incubation at 4 °C with that of cells kept under normal 37 °C conditions. The cell membranes were stained, after which the cells were exposed to 100 nm nanoparticles for 1 h, fixed, and lastly observed using confocal microscopy. The panels show example images of nanoparticles (cyan) and the outer cell membrane (CellMask; red). **(a)** Cells under normal conditions, *i.e.*, exposed to particles at 37 °C. White arrows indicate particles that are inside the cell. **(b–c)** Cells subjected to prior incubation at 4 °C. Cells were kept at 4 °C for 30 min to shut down energy-dependent mechanisms. They were then maintained at 4 °C during the 1 h nanoparticle exposure, prior to fixation. A few particles adsorbed to the outer cell membrane or regions between cells may be observed, but, importantly, no particles can be seen within the cells. This is consistent with the particles being internalized *via* a cell energy dependent mechanism. Scale bars 10 µm.

Nanoparticle uptake mechanism assessed with pharmacological inhibitors

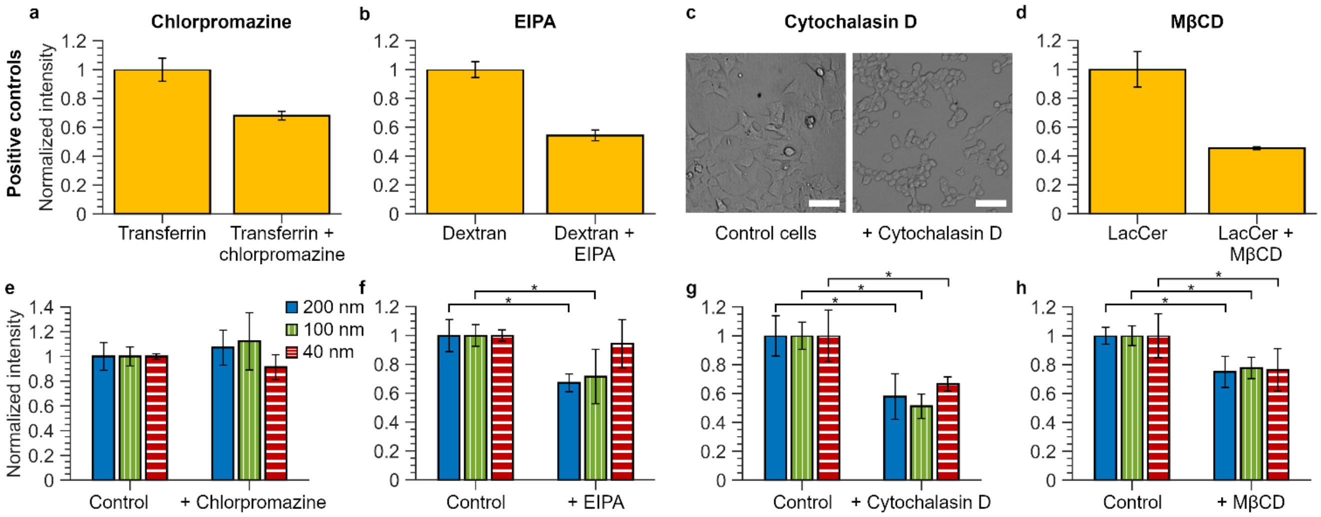

Supporting Figure S9. Nanoparticle uptake in the presence of pharmacological inhibitors of endocytic pathways in live HEK cells. A panel of four pharmacological inhibitors was applied to assess which entry mechanisms contributed to nanoparticle uptake in our studies: chlorpromazine was used to inhibit clathrin-mediated endocytosis; EIPA to inhibit macropinocytosis, cytochalasin D to inhibit actin polymerization, and MβCD to deplete cholesterol.[9–13] **(a–d)** Positive controls to confirm that inhibition of the various mechanisms was achieved. **(a)** Transferrin cellular uptake with and without chlorpromazine. **(b)** Dextran cellular uptake with and without EIPA. **(c)** Phase gradient contrast imaging to confirm that cytochalasin D treatment prevented actin polymerization leading to a rounded cell morphology. **(d)** LacCer cellular uptake with and without MβCD. In all cases the positive controls indicated that the inhibitors were functioning. In the case of the bar plots, the results are the mean of 3 repeats and error bars are the standard deviations. **(e–h)** Nanoparticle uptake in the absence (control) and presence of the various inhibitors for 200 nm, 100 nm and 40 nm nanoparticles. **(e)** Chlorpromazine treatment to inhibit clathrin-mediated endocytosis resulted in an increase in particle uptake compared to controls for 200 nm and 100 nm particles, whereas 40 nm particles showed a small (non-significant) decrease in uptake. **(f)** Inhibition of macropinocytosis resulted in reduced uptake of 200 nm particles and, with a higher variability, 100 nm particles, whereas macropinocytosis did not appear to contribute strongly to 40 nm particle uptake by HEK cells. **(g)** Inhibition of actin polymerization with cytochalasin D resulted in reduced uptake for all particle sizes. **(h)** Cholesterol depletion reduced uptake of all particle sizes to a minor extent. Results are the mean of multiple experiments, each performed in triplicate and error bars represent the standard deviation over all replicates. Asterisks indicate a statistically significant ($p < 0.05$) lowering of particle uptake by the inhibitor-treated cells compared to control.

Nanoparticle colocalization with lysosomes

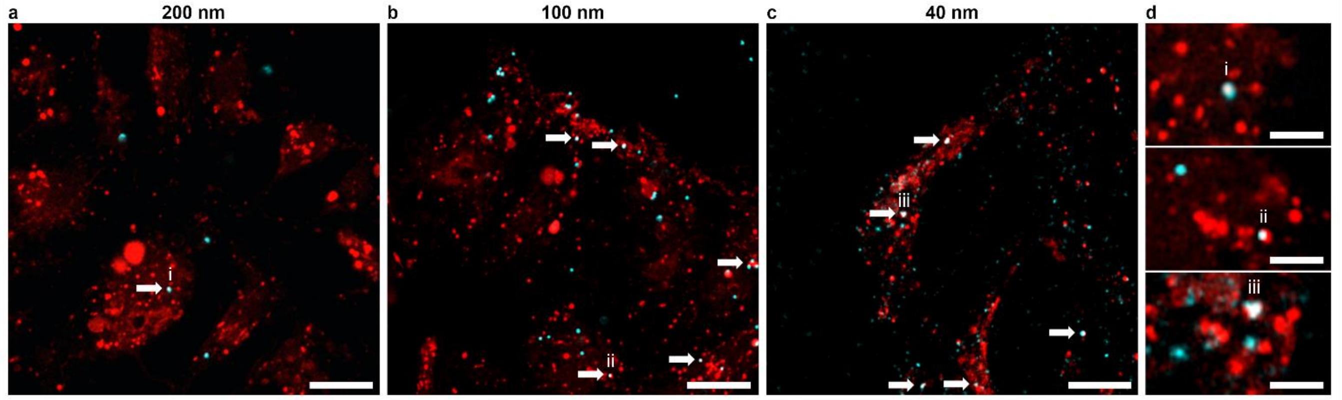

Supporting Figure S10. Nanoparticle colocalization with lysosomes in live HEK cells. For a range of nanoparticle/cell systems, it has been shown that particles that enter cells through endocytosis are trafficked through the endolysosomal pathway,[3,13–17] though non-endolysosomal pathways have also been implicated.[15,18] To determine the extent of endolysosomal trafficking for our system, we used live-cell confocal microscopy to visualize internalized particles and lysosomes stained with LysoTracker. HEK cells were incubated with nanoparticles for 30 min (pulse), washed, and then left to internalize the particles for 4 h (chase). Confocal microscopy images of cells incubated with **(a)** 200 nm particles, **(b)** 100 nm particles and **(c)** 40 nm particles (cyan) with stained lysosomes (red). In all cases, some particles were colocalized with lysosomes yielding a white signal in the particle-lysosome overlay (examples indicated with white arrows), whereas many other particles were not colocalized with lysosomes. Scalebars 50 µm. **(d)** Zoom in of the colocalized particles denoted with i, ii, and iii, respectively, in panels a–c. Scalebars 3 µm.

Supporting Table S4. Nanoparticle colocalization with lysosomes in live HEK cells. Confocal microscopy was used to assess the colocalization of nanoparticles with lysosomes after 30 min exposure followed by 4 h incubation. Lysosomes were stained with LysoTracker and several images of multiple cells such as those shown in Supporting Figure S10 were used. Colocalization was then determined by identifying internalized particles with overlapping peaks in the nanoparticle (yellow/green emission) and the LysoTracker (orange/red emission) channels. The number of particles colocalized with lysosomes and the total number of particles within the cell boundaries, *N*, were used to calculate the percentage of particles within lysosomes for each particle size. For all nanoparticle sizes investigated, many particles were trafficked to the lysosomes. However, a substantial proportion did not colocalize with lysosomes. This observation is consistent with previous reports in other cells lines.[3,16]

Particle	N	Percentage of particles in lysosomes
200 nm	38	32 %
100 nm	130	39 %
40 nm	379	31 %

Membrane envelopment of internalized particles

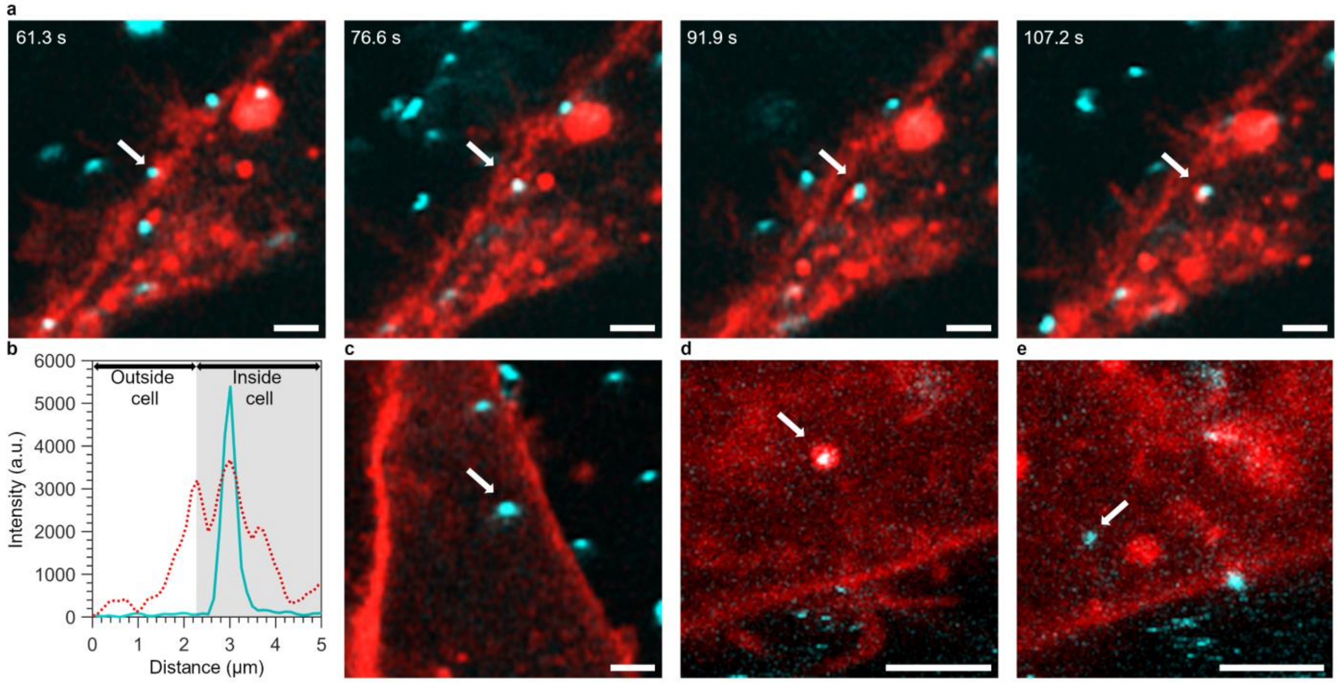

Supporting Figure S11. Nanoparticle colocalization with cell membrane stain-labelled structures in live HEK cells. For some of the internalization events identified in this work, we visualized an accompanying cell membrane stain (CellMask or Abberior Star 580-DPPE for the confocal and STED microscopy experiments, respectively) labelled membrane enveloping the particle whilst entering the cell, presumably the endocytic vesicle associated with the internalization process (Figure 1e). However, in many cases we did not observe this (Supporting Videos S1-S3). Here we give further examples of both observations. **(a)** Time-lapse confocal microscopy images of the internalization of a 100 nm particle (cyan) by CellMask-stained cells (red). The particle of interest is denoted with white arrows. The particle is adsorbed to the plasma membrane for 61.3 s after which it is internalized at t = 76.6 s. At this timepoint the particle signal overlaps with a CellMask-stained structure which extends from the plasma membrane towards the interior of the cell. At t = 91.9 s and t = 107.2 s the particle travels further within the cell and appears to be accompanied by the CellMask-stained structure, presumably a vesicle. **(b)** Intensity profile along a line of the particle shown in panel a at t = 76.6 s. The particle intensity is given by the solid cyan line, the CellMask intensity is given by the dotted red line, and the inside of cell is denoted by the light gray shading. The first peak in the CellMask intensity profile is the plasma membrane. The particle intensity peak coincides with a second peak in the CellMask signal, likely a vesicle. **(c)** Confocal microscopy image of a 100 nm particle clearly within the interior of the cell which is not accompanied by a CellMask-stained vesicle. **(d, e)** STED images of 40 nm internalized particles (indicated by white arrows) either **(d)** colocalized with an Abberior Star 580-DPPE-stained structure or **(e)** not colocalized. Scalebars 2 µm. Overall, we mostly observed particles entering without an associated cell membrane stain-labelled structure. However, we observed several examples where an accompanying cell membrane stain-labelled structure, presumably a vesicle, could be visualized (*e.g.*, 7 of 45 observed internalization events for the 100 nm particles in the long-term imaging experiments). Moreover, some particles for which we did not see the entry process, but which were clearly within the cell, were enveloped by a cell membrane stain-labelled structure but, again, many others were not.

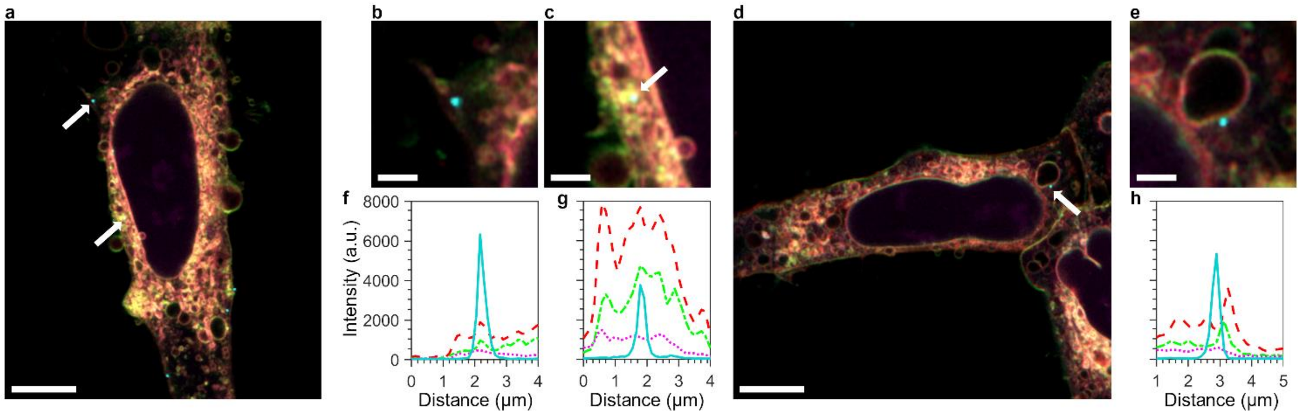

Supporting Figure S12. Nanoparticle colocalization with Nile red staining in live HEK cells. Some internalized particles colocalized with cell membrane stain (CellMask or Abberior Start 580-DPPE) labelled structures (Figure 1e and Supporting Figure S11), whereas other particles entered without an associated cell membrane stain-labelled structure. This could be due to either insufficient inclusion of cell membrane stain in some of the cell membrane-derived vesicles, due to some particles being internalized without an enveloping membrane, or due to some particles having progressed to non-cell membrane stain-labelled structures intracellularly. To shed some light on this matter, we used Nile red to stain all internal membranes and lipid structures, as well as the plasma membrane. Thereby, we could assess whether internalized particles were enclosed in membrane structures, regardless of the exact nature of the organelle. The emission spectrum of Nile red shifts depending on the polarity of its environment, which differs for various organelles.[19] Thus we used green/yellow, orange/red and far red emission to image the Nile red staining and used 100 nm blue nanoparticles (which show no crosstalk with the broad Nile red emission). Cells were incubated with particles for 30 min followed by washing, fixation and then Nile red staining. In this manner we could assess particle-membrane colocalization within the early stages after particle internalization. **(a–e)** Confocal microscopy images of cells incubated with 100 nm particles (cyan) and Nile red stained structures, observed using yellow/green (green), orange/red (red) and far red (magenta) emission spectrum. **(a, d)** Images of entire cells where nanoparticles can be visualized within cells as well as on the plasma membrane. White arrows indicate examples of internalized particles. Scalebars 10 µm. **(b, c, e)** Zoom in images of the particles indicated with white arrows in panels a and d. Scalebars 2 µm. **(f–h)** Intensity profiles of the particles shown in panels b, c, and e, respectively, along a line. The particle signal is given by the solid cyan line whereas the other three profiles denote the Nile red signal from the different emission spectra measured (yellow/green emission: dot-dash green line, orange/red emission: dash red line, and dark red emission: dot magenta line). 28 of the 30 identified internalized particles colocalized with peaks in the Nile red staining (panels a, b, f, g). However, 2 out of 30 particles did not colocalize with internalized membranes. An example of the latter is shown in panels e and h where the particle can be clearly seen on the outside of a large membrane bound structure. We cannot exclude that these 2 particles entered without an endocytic vesicle, though it would seem unlikely as particle uptake was completely suppressed when cell energy was depleted (Supporting Figure S8). Alternatively, these particles may have escaped from membrane structures and into the cytosol after uptake, though further studies would be required to confirm this. Nevertheless, we conclude that the vast majority of particles enter cells *via* vesicles, and consequently that the observation of rapid internalization (Figure 3) is unrelated to the presence or absence of an observed enveloping membrane.

STED imaging parameters

Figure 1c and Supporting Figure S11d–e

640 nm laser settings: 4%, 600 ps delay, 12 ns width => 4% STED
560 nm laser settings: 23%, 600 ps delay, 12 ns width => 6% STED
Two line steps for both channels, and a pixel dwell time of 10 µs
Pinhole setting: 1.0 AU
Field of view: 45 nm pixels, 52 × 224 pixels (2.340 µm × 11.648 µm)

Figure 1d

640 nm laser settings: 2%, 600 ps delay, 12 ns width => 4% STED
560 nm laser settings: 13%, 600 ps delay, 12 ns width => 6% STED
Two line steps for both channels, and a pixel dwell time of 10 µs
Pinhole setting: 1.0 AU
Field of view: 45 nm pixels, 131 × 353 pixels (5.895 µm × 15.885 µm)

Figure 1e

640 nm laser settings: 5%, 600 ps delay, 12 ns width => 4% STED
560 nm laser settings: 23%, 600 ps delay, 12 ns width => 6% STED
Three line steps for the particle channel and two line steps for the membrane channel, and a pixel dwell time of 5 µs
Pinhole setting: 1.0 AU
Field of view: 45 nm pixels, 231 × 313 pixels (10.395 µm × 14.085 µm)

Figure 3f and Supporting Video S5

640 nm laser settings: 2%, 600 ps delay, 12 ns width => 3% STED
560 nm laser settings: 10%, 600 ps delay, 12 ns width => 4% STED
Two line steps for both channels, and a pixel dwell time of 10 µs
Pinhole setting: 1.0 AU
Field of view: 45 nm pixels, 264 × 112 pixels (11.880 µm × 5.040 µm)

Supporting videos

Supporting Video S1

40 nm yellow-green particles (cyan) added to plasma membrane stained HEK cells (red) and imaged using confocal microscopy. Arrow denotes a particle of interest that can be seen to adsorb to the plasma membrane and is then internalized into the cell (15.33 s per frame). Background subtraction has been performed as described in the Materials and Methods. Snap shot depicted.

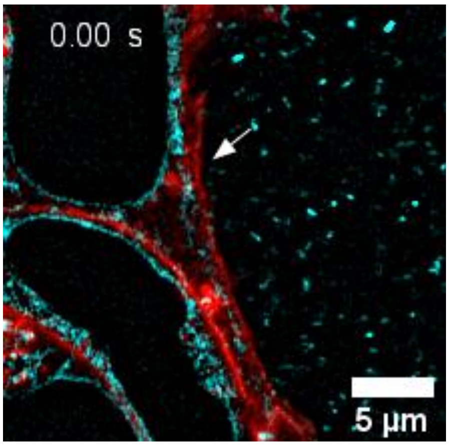

Supporting Video S2

100 nm yellow-green particles (cyan) added to plasma membrane stained HEK cells (red) and imaged using confocal microscopy. Arrow denotes a particle of interest that can be seen to adsorb to the plasma membrane and is then internalized into the cell (15.11 s per frame). Snap shot depicted.

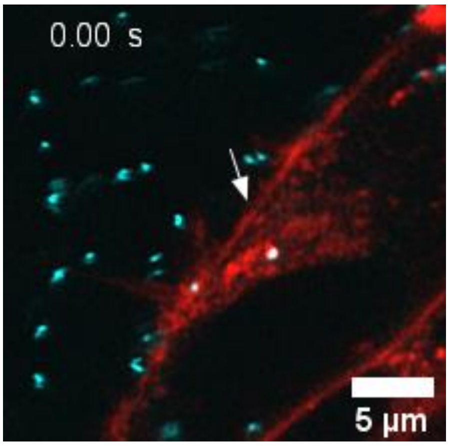

Supporting Video S3

200 nm yellow-green particles (cyan) added to plasma membrane stained HEK cells (red) and imaged using confocal microscopy. Arrow denotes a particle of interest that can be seen to adsorb to the plasma membrane and is then internalized into the cell (15.24 s per frame). Snap shot depicted.

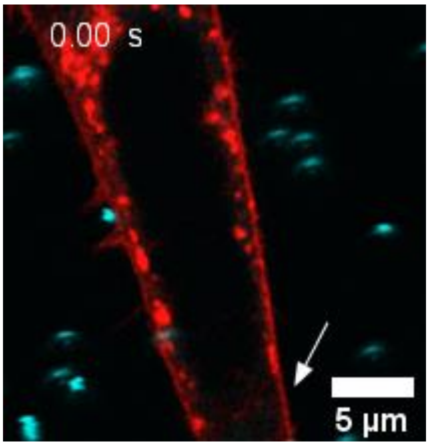

Supporting Video S4

100 nm yellow-green particles (cyan) added to plasma membrane stained HEK cells (red) and imaged using confocal microscopy. Arrow denotes a particle of interest that can be seen to adsorb to the plasma membrane and is then internalized into the cell after just a 1.25 s. The particle then transports further into the cell (1.25 s per frame). Snap shot depicted.

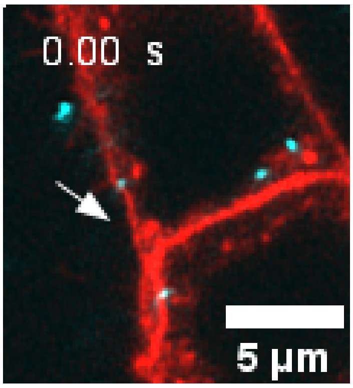

Supporting Video S5
40 nm dark red particles (cyan) added to plasma membrane stained HEK cells (red) and imaged using STED microscopy. Arrow denotes a particle of interest that can be seen to adsorb to the plasma membrane and is then internalized into the cell after just a few seconds (~1 s per frame). Snap shot depicted.

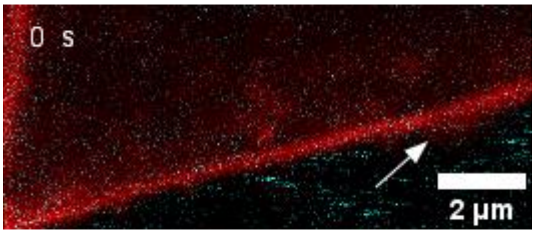

Supporting Video S6
100 nm yellow-green particles (cyan) added to plasma membrane stained HEK cells (red) and imaged using confocal microscopy. Arrow denotes a particle of interest that can be seen to adsorb to the plasma membrane and is then internalized into the cell (1.33 s per frame). The particle can no longer be observed in the timepoint immediately after internalization. Snap shot depicted.

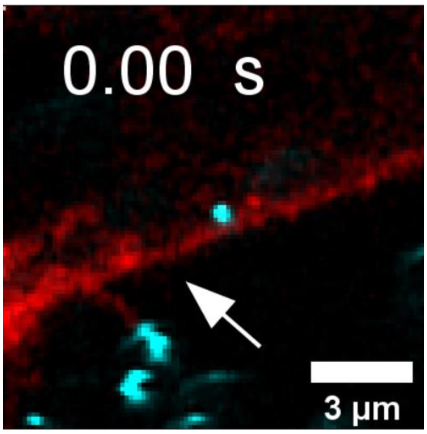

References

1. Lesniak, A. *et al.* Nanoparticle adhesion to the cell membrane and its effect on nanoparticle uptake efficiency. *J. Am. Chem. Soc.* **135**, 1438–1444 (2013).
2. Varela, J. A., Bexiga, M. G., Åberg, C., Simpson, J. C. & Dawson, K. A. Quantifying size-dependent interactions between fluorescently labeled polystyrene nanoparticles and mammalian cells. *J. Nanobiotech.* **10**, 39 (2012).
3. Vtyurina, N., Åberg, C. & Salvati, A. Imaging of nanoparticle uptake and kinetics of intracellular trafficking in individual cells. *Nanoscale* **13**, 10436–10446 (2021).
4. de Boer, I., Richards, C. J. & Åberg, C. Simultaneous exposure of different nanoparticles influences cell uptake. *Pharmaceutics* **14**, 136 (2022).
5. Kim, J. A., Salvati, A., Åberg, C. & Dawson, K. A. Suppression of nanoparticle cytotoxicity approaching in vivo serum concentrations: limitations of in vitro testing for nanosafety. *Nanoscale* **6**, 14180–14184 (2014).
6. Ye, D. *et al.* Low uptake of silica nanoparticles in Caco-2 intestinal epithelial barriers. *Beilstein J. Nanotech.* **8**, 1396–1406 (2017).
7. Åberg, C., Varela, J. A., Fitzpatrick, L. W. & Dawson, K. A. Spatial and structural metrics for living cells inspired by statistical mechanics. *Sci. Rep.* **6**, 34457 (2016).
8. Salvati, A. *et al.* Experimental and theoretical comparison of intracellular import of polymeric nanoparticles and small molecules: toward models of uptake kinetics. *Nanomedicine Nanotech. Biol. Med.* **7**, 818–826 (2011).
9. Francia, V., Reker-Smit, C., Boel, G. & Salvati, A. Limits and challenges in using transport inhibitors to characterize how nano-sized drug carriers enter cells. *Nanomedicine* **14**, 1533–1549 (2019).
10. Francia, V. *et al.* Corona composition can affect the mechanisms cells use to internalize nanoparticles. *ACS Nano* **13**, 11107–11121 (2019).
11. Jiang, X. *et al.* Endo- and exocytosis of zwitterionic quantum dot nanoparticles by live hela cells. *ACS Nano* **4**, 6787–6797 (2010).
12. Jiang, X. *et al.* Specific effects of surface carboxyl groups on anionic polystyrene particles in their interactions with mesenchymal stem cells. *Nanoscale* **3**, 2028–2035 (2011).
13. Yang, L., Shang, L. & Nienhaus, G. U. Mechanistic aspects of fluorescent gold nanocluster internalization by live HeLa cells. *Nanoscale* **5**, 1537–1543 (2013).
14. Iversen, T.-G., Skotland, T. & Sandvig, K. Endocytosis and intracellular transport of nanoparticles: Present knowledge and need for future studies. *Nano Today* **6**, 176–185 (2011).
15. Sandin, P., Fitzpatrick, L. W., Simpson, J. C. & Dawson, K. A. High-speed imaging of Rab family small GTPases reveals rare events in nanoparticle trafficking in living cells. *ACS Nano* **6**, 1513–1521 (2012).
16. Vercauteren, D. *et al.* Dynamic colocalization microscopy to characterize intracellular trafficking of nanomedicines. *ACS Nano* **5**, 7874–7884 (2011).
17. Reinholz, J. *et al.* Protein machineries defining pathways of nanocarrier exocytosis and transcytosis. *Acta Biomaterialia* **71**, 432–443 (2018).
18. Lai, S. K. *et al.* Privileged delivery of polymer nanoparticles to the perinuclear region of live cells via a non-clathrin, non-degradative pathway. *Biomat.* **28**, 2876–2884 (2007).
19. Zhanghao, K. *et al.* High-dimensional super-resolution imaging reveals heterogeneity and dynamics of subcellular lipid membranes. *Nat. Commun.* **11**, 5890 (2020).
